# Label-free morphology-based phenotypic analysis of spinal and bulbar muscular atrophy muscle cell models

**DOI:** 10.1242/dmm.052220

**Published:** 2025-06-25

**Authors:** Kenji Sakakibara, Kenjiro Tanaka, Madoka Iida, Yuta Imai, Mai Okada, Kentaro Sahashi, Tomoki Hirunagi, Kentaro Maeda, Ryuji Kato, Masahisa Katsuno

**Affiliations:** ^1^ Department of Neurology, Nagoya University Graduate School of Medicine, Tokai National Higher Education and Research System, 65 Tsurumai-cho, Showa-ku, Nagoya, Aichi 466-8550, Japan; ^2^ Department of Basic Medicinal Sciences, Graduate School of Pharmaceutical Sciences, Nagoya University, Tokai National Higher Education and Research System, Furocho, Chikusa-ku, Nagoya, Aichi 464-8601, Japan; ^3^ Nagoya University Institute for Advanced Research, Furo-cho, Chikusa-ku, Nagoya, Aichi 464-8601, Japan; ^4^ Institute of Nano-Life-Systems, Institutes of Innovation for Future Society, Nagoya University, Tokai National Higher Education and Research System, Furocho, Chikusa-ku, Nagoya, Aichi 464-8601, Japan; ^5^ Institute for Glyco-core Research (iGCORE), Tokai National Higher Education and Research System, Furocho, Chikusa-ku, Nagoya, Aichi 464-8601, Japan

**Keywords:** Spinal and bulbar muscular atrophy, Morphology-based phenotypic analysis, Muscle cell models, Naratriptan

## Abstract

Spinal and bulbar muscular atrophy (SBMA) is a neuromuscular disorder caused by CAG trinucleotide expansion in the androgen receptor (*AR*) gene. To improve the quality of *in vitro* cell-based assays for the evaluation of potential drug candidates for SBMA, we developed a morphology-based phenotypic analysis for a muscle cell model of SBMA that involves multiparametric morphological profiling to quantitatively assess the therapeutic effects of drugs on muscle cell phenotype. The analysis was validated using dihydrotestosterone and pioglitazone, which have been shown to exacerbate and ameliorate the pathophysiology of SBMA, respectively. Gene expression analysis revealed activation of the JNK pathway in the SBMA cells compared to the control cells. Phenotypic analysis revealed the effect of naratriptan, a JNK inhibitor, on the phenotypic changes of SBMA cells, and the results were confirmed by LDH assays. We then trained a predictive machine learning model to classify the drug responses, and it successfully discriminated between pioglitazone-type and naratriptan-type morphological profiles based on their morphological characteristics. Our morphology-based phenotypic analysis provides a noninvasive and efficient screening method to accelerate the development of therapeutics for SBMA.

## Teaser

**Summary:** This study established a noninvasive and efficient *in vitro* drug screening technique based on morphology-based phenotypic analysis using a muscle cell model of spinal and bulbar muscular atrophy.

## INTRODUCTION

Spinal and bulbar muscular atrophy (SBMA) is an adult-onset, X-linked neuromuscular disease caused by an abnormal expansion of CAG repeats in the androgen receptor (*AR*) gene. An increase in the number of CAG repeats leads to the repetition of glutamic acid in ARs, resulting in abnormal AR aggregation in the nucleus and ultimately leading to cell death (Katsuno et al., 2012; Sobue et al., 1989; Kennedy et al., 1968).

Previous studies have revealed that the pathogenesis of SBMA involves not only neurogenic but also myogenic changes. Understanding the mechanisms of muscle dysfunction is a crucial aspect of SBMA research. Patients with SBMA are known to exhibit elevated levels of creatine kinase in serum, and their skeletal muscle biopsies display both neurogenic and myogenic changes (Sorarù et al., 2008). The mutant AR suppresses the transcription of the *SLC6A8* gene, which encodes a creatine transporter, impeding creatine uptake into muscle cells (Hijikata et al., 2016). Skeletal muscle pathology was found to precede neurodegeneration in an animal model of SBMA (Yu et al., 2006). Furthermore, muscle-specific knockout of the mutant AR improved motor neuron phenotypes in a mouse model of SBMA (Cortes et al., 2014), suggesting an alternative therapeutic approach targeting the skeletal muscles of SBMA. Given the limited availability of effective treatments for SBMA, there is a growing need for a deeper understanding of its underlying mechanisms, particularly within the context of muscle dysfunction.

Traditional procedures for assessing the pathology of cells and the efficacy of potential candidate drugs have several limitations. Differences between conditions are often difficult to detect in assay methods that measure end-point readouts, such as absorbance or fluorescence emission. Cells need to be labeled with reagents, and they cannot be reused for other assays. With respect to myocytes, cell elongation has been thought to represent myotube formation, and morphological analysis of myocytes from disease models has traditionally focused on intentionally defined features such as cell length and cell width (Malena et al., 2013; Milioto et al., 2017). However, subjective morphological analysis is limited in terms of the number of cells that can be analyzed and tends to subjectively evaluate selected partial cellular changes. Furthermore, morphological changes can occur in a variety of circumstances, including the apoptosis of myotubes (D'Emilio et al., 2010; Häcker, 2000). Based on these insights, it is emphasized that relying solely on limited morphological features may mislead interpretations.

In contrast to conventional invasive bulk assays, image analysis provides a highly efficient and comprehensive data acquisition approach capable of capturing real-time and time-course responses, particularly with respect to cellular heterogeneity. In addition, to enhance the objectivity of label-free morphological analysis, our research group has performed a comprehensive examination of multiple morphological parameters over time to characterize heterogeneous responses within cell populations. This approach has demonstrated superior and robust performance in predicting the qualities of various cell types, including mesenchymal stem cells (Imai et al., 2018; Sasaki et al., 2014, 2016; Takemoto et al., 2021), induced pluripotent cells (Kato et al., 2016), neuronal cells (Fujitani et al., 2017; Kawai et al., 2016) and myoblasts (Ishikawa et al., 2019). In our previous research, we applied a morphological analysis approach to a neuronal cell model of SBMA and demonstrated that morphological phenotypic profiles serve as important indicators for assessing the efficacy of drugs in a neuronal cell model of SBMA (Imai et al., 2022).

In the present study, we applied comprehensive morphology-based phenotypic analysis to a muscle cell model of SBMA generated from C2C12 myoblast cells. We aimed to explore the potential of morphology-based phenotypic analysis to detect the therapeutic effects of drugs using image profiles. After validating the analysis using dihydrotestosterone (DHT) and pioglitazone (PG), we tested the efficacy of five potential compounds in the SBMA cell model. We identified new important pathways involved in the muscular pathogenesis of SBMA from the image profile analysis.

## RESULTS

### Morphology-based phenotypic analysis can be used to quantitatively evaluate the effects of positive control drugs in a muscle cell model of SBMA

**Fig. 1.**
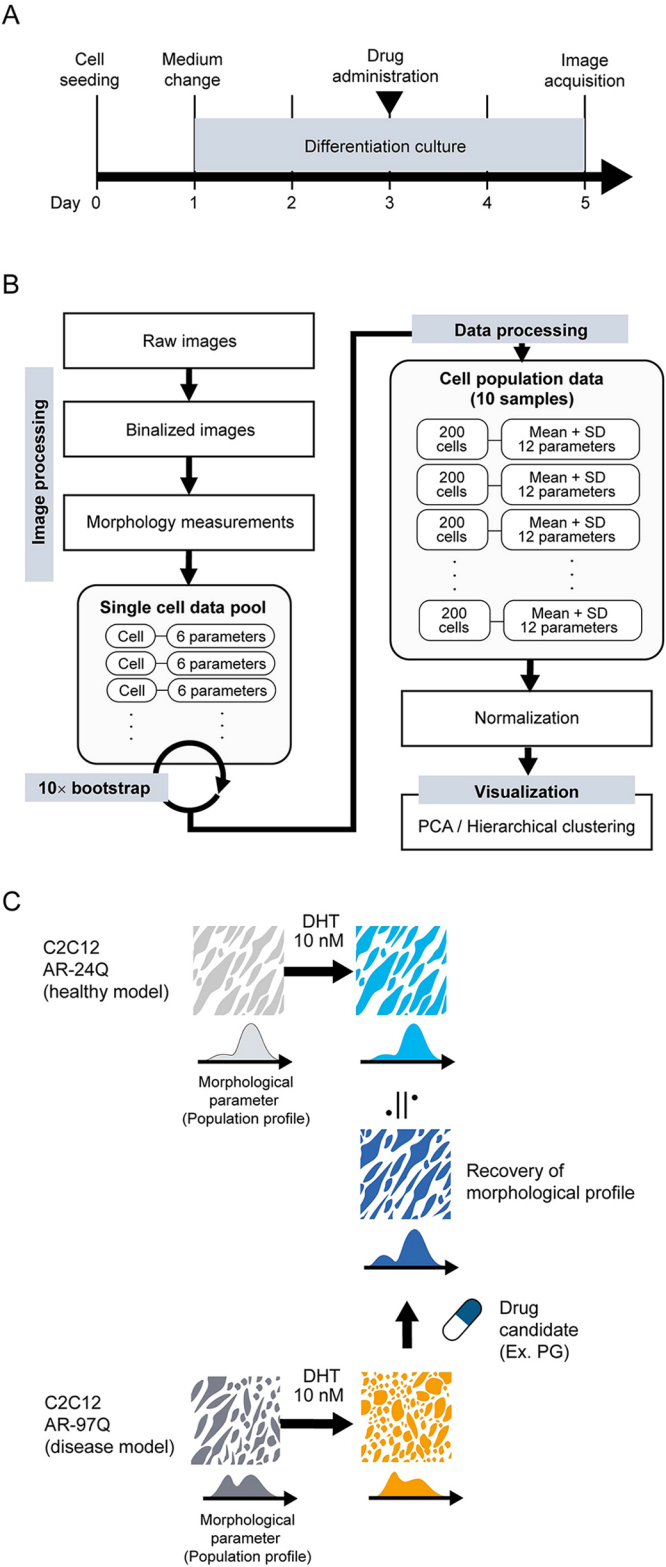
**Schematic illustration of the label-free morphology-based phenotypic analysis for analyzing myoblast spinal and bulbar muscular atrophy (SBMA) model cells.** (A) Experimental timeline. (B) Image and data processing pipeline. (C) Concept of morphology-based phenotypic analysis of drug effects utilizing C2C12 cell models: AR-24Q cells and AR-97Q cells. Drug candidates induce the recovery of AR-97Q cells from a disease state to a nondisease state based on morphological observation. DHT, dihydrotestosterone; PCA, principal component analysis; PG, pioglitazone; SD, standard deviation.

To establish a morphology-based phenotypic analysis method for a muscle cell model of SBMA, we fine-tuned the image processing pipeline originally developed to assess the responses of C2C12 cells to myotube formation (Ishikawa et al., 2019). We validated its ability to assess the cell model of SBMA according to the experimental scheme (Fig. 1A). Cells stably expressing AR with 24 CAG repeats (AR-24Q cells) were considered a nondisease control model, and cells stably expressing AR with 97 CAG repeats (AR-97Q cells) were considered a cell model of SBMA (Iida et al., 2019). Phase-contrast microscopy images were taken for the evaluation of cells after 4 days of myotube differentiation, and the morphological profiles of the total cell population were extracted as readouts from the image processing (Fig. 1B). The analysis involved a two-step processing pipeline. First, each cell was annotated with six morphological parameters (area, perimeter, length, width, length-to-width ratio and compactness) (Table S1). From the single-cell data pool, a representative cell population dataset was generated by randomly selecting 200 single-cell data using bootstrap sampling. Subsequently, each cell population was characterized by 12 statistical values, the mean and standard deviation of each morphological parameter, as detailed in Table S1. These values collectively constituted the population's ‘morphological profile’. Then, the morphological profiles of AR-24Q and AR-97Q cells treated with or without drug candidates were compared, and the similarities in their profiles were analyzed to determine the therapeutic effects of the drugs (Fig. 1C).

**Fig. 2.**
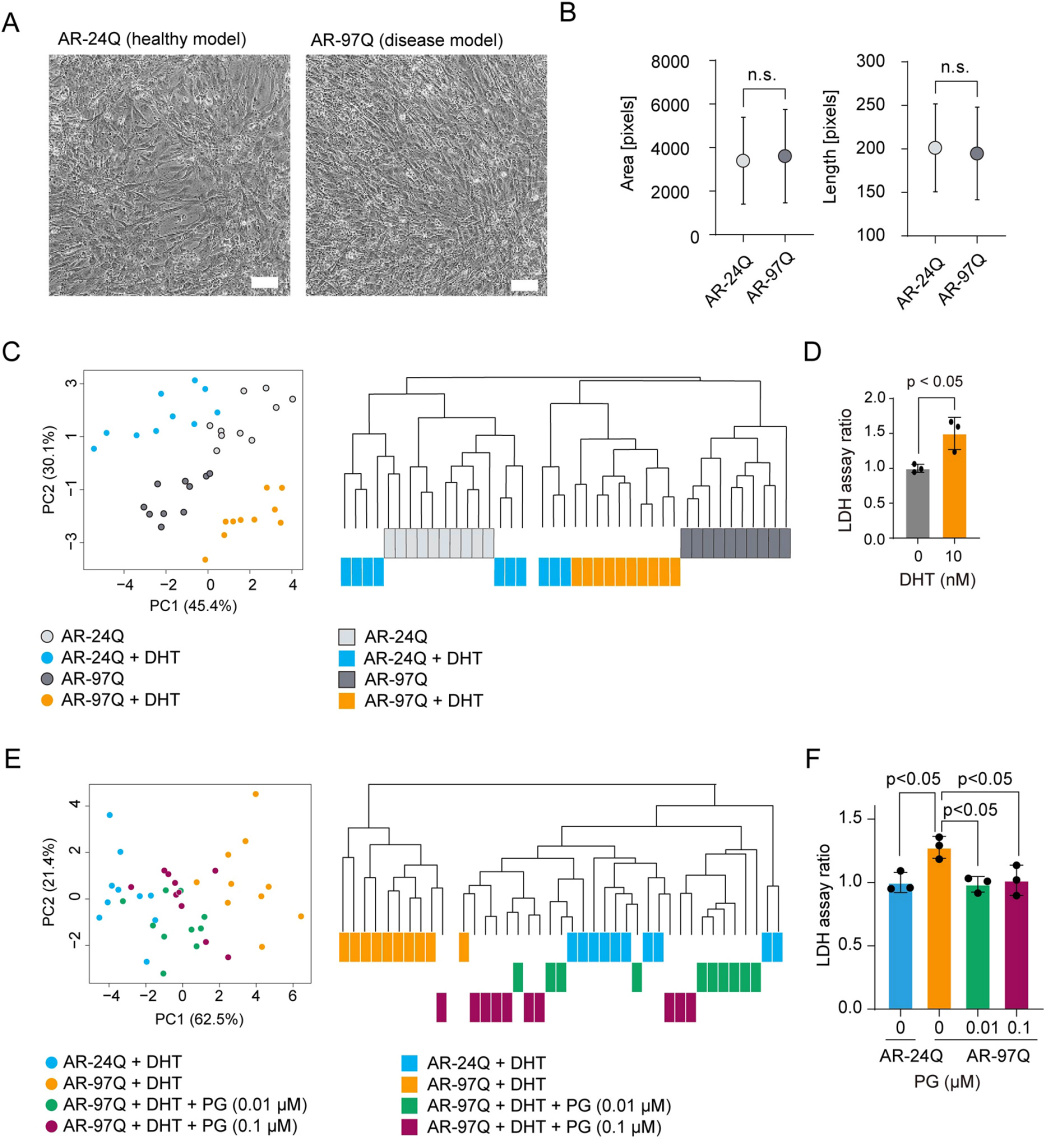
**Morphology-based phenotypic analysis can be used to quantitatively evaluate drug efficacy in a muscle cell model of SBMA.** (A) Representative images of C2C12 cells stably expressing full-length human ARs containing 24 (right) or 97 (left) CAG repeats that were treated with 10 nM dihydrotestosterone (DHT). Scale bars: 200 µm. (B) Morphological distribution of 300 cells measured by a single parameter (*N*=300). (C) PCA and hierarchical clustering of morphological profiles obtained from phase-contrast images of AR-24Q or AR-97Q cells that were treated with or without DHT. PC, principal component. (D) Lactate dehydrogenase (LDH) release from AR-97Q cells treated with or without DHT. (E) PCA and hierarchical clustering of morphological profiles obtained from phase-contrast images of AR-24Q or AR-97Q cells treated with or without pioglitazone (PG). (F) LDH release from AR-24Q and AR-97Q cells treated with DHT. The AR-97Q cells were treated with or without PG. Quantitative analyses were performed on *N*=3 samples per group. Error bars indicate s.d. Statistical analysis with unpaired two-sided *t*-tests (B,D) or one-way ANOVA with post hoc Dunnett's test (F). n.s., not significant.

Compared with AR-24Q cells, AR-97Q cells had a slightly greater prevalence of random alignment of elongated cells, but this difference was difficult to detect under a conventional microscope (Fig. 2A). Assessment of cell size using single morphological parameters such as area and length, which are commonly used to analyze cell size, also proved insufficient to distinguish morphological differences between AR-24Q and AR-97Q cells (Fig. 2B). Although morphological parameters such as area, length and width are recognized as indicators of myotube formation, subjectively selected single morphological parameters are insufficient for evaluating the morphological characteristics of C2C12 cells. A comparison of the levels of major histocompatibility complex (MHC), a myogenic marker frequently utilized to identify the differentiation stages, also revealed no significant disparities between the AR-24Q and AR-97Q cells (Fig. S1). Based on these findings, 12 morphological parameters (six parameters, 12 statistical values) (Table S1) were used in our morphology-based phenotypic analysis, and principal component analysis (PCA) and hierarchical clustering revealed distinctions between the cell types (Fig. 2C). Because principal component (PC)1 in PCA was dominated by the standard deviations of morphological parameters, PCA indicated that AR-24Q cells form more homogeneous myotubes upon DHT stimulation, whereas AR-97Q cells exhibit greater variability, suggesting disorganized differentiation. PC2 was weighted by myotube width, indicating that AR-24Q cells tend to form thicker, more mature myotubes compared to those formed by AR-97Q cells. The clustering branches not only highlighted the differences between AR-24Q and AR-97Q cells but also effectively distinguished between AR-97Q cells treated with or without DHT. The uniformity observed within each cluster indicates that the morphological profiles remain consistent, despite their statistical profiles being derived from different individual cells. Lactate dehydrogenase (LDH) assay was used to measure cellular damage, and the results showed that LDH release was significantly greater in AR-97Q cells treated with DHT than in AR-97Q cells not treated with DHT (Fig. 2D), suggesting that the quantified morphological phenotypes accurately reflect disease-related cellular damage. Thus, morphology-based phenotypic analysis was confirmed to be useful for differentiating SBMA model C2C12 cells from control cells.

We further validated our morphology-based phenotypic analysis by administering PG to AR-97Q cells treated with DHT. PG acts as an activator of peroxisome proliferator-activated receptor-γ (PPARγ) and reportedly ameliorates the pathogenesis of motor neurons and skeletal muscles in SBMA by inhibiting oxidative stress, suppressing nuclear factor kappa B (NFκB) signaling and reducing inflammatory responses in a mouse model of SBMA (AR-97Q) (Iida et al., 2015). Thus, it was expected that the administration of PG to AR-97Q cells would restore the phenotypes of the cells. The protein levels of PPARγ in AR-97Q cells were found to be lower than those in AR-24Q cells. Moreover, the administration of 0.01 or 0.1 μM PG to AR-97Q cells resulted in an increase in PPARγ levels, which was consistent with the findings of a previous report (Fig. S2) (Iida et al., 2015). The introduction of PG induced a morphological change in AR-97Q cells to resemble the morphology of AR-24Q cells (Fig. 2E). Furthermore, new clusters of AR-97Q cells treated with PG were clustered within the same branch of AR-24Q cells according to hierarchical clustering (Fig. 2E). The restorative effect of PG on DHT-treated AR-97Q cells observed in the phenotypic analysis was consistent with the results of a previous report that demonstrated the efficacy of PG in a muscle cell model of SBMA (Iida et al., 2015). LDH assays revealed a significant reduction in the toxicity of DHT-treated AR-97Q cells by treatment with PG, with LDH released reduced to levels comparable to those released from AR-24Q cells in response to 0.1 μM PG (Fig. 2F). These findings suggested that the quantified morphological phenotypes accurately reflect the amelioration of SBMA model C2C12 cell pathology. This experiment confirmed the efficacy of our morphology-based phenotypic analysis in detecting the therapeutic effects of compounds on AR-97Q cells.

### Identification of disease-associated pathways via comparative analysis of expression profiles between AR-24Q and AR-97Q cells

**Fig. 3.**
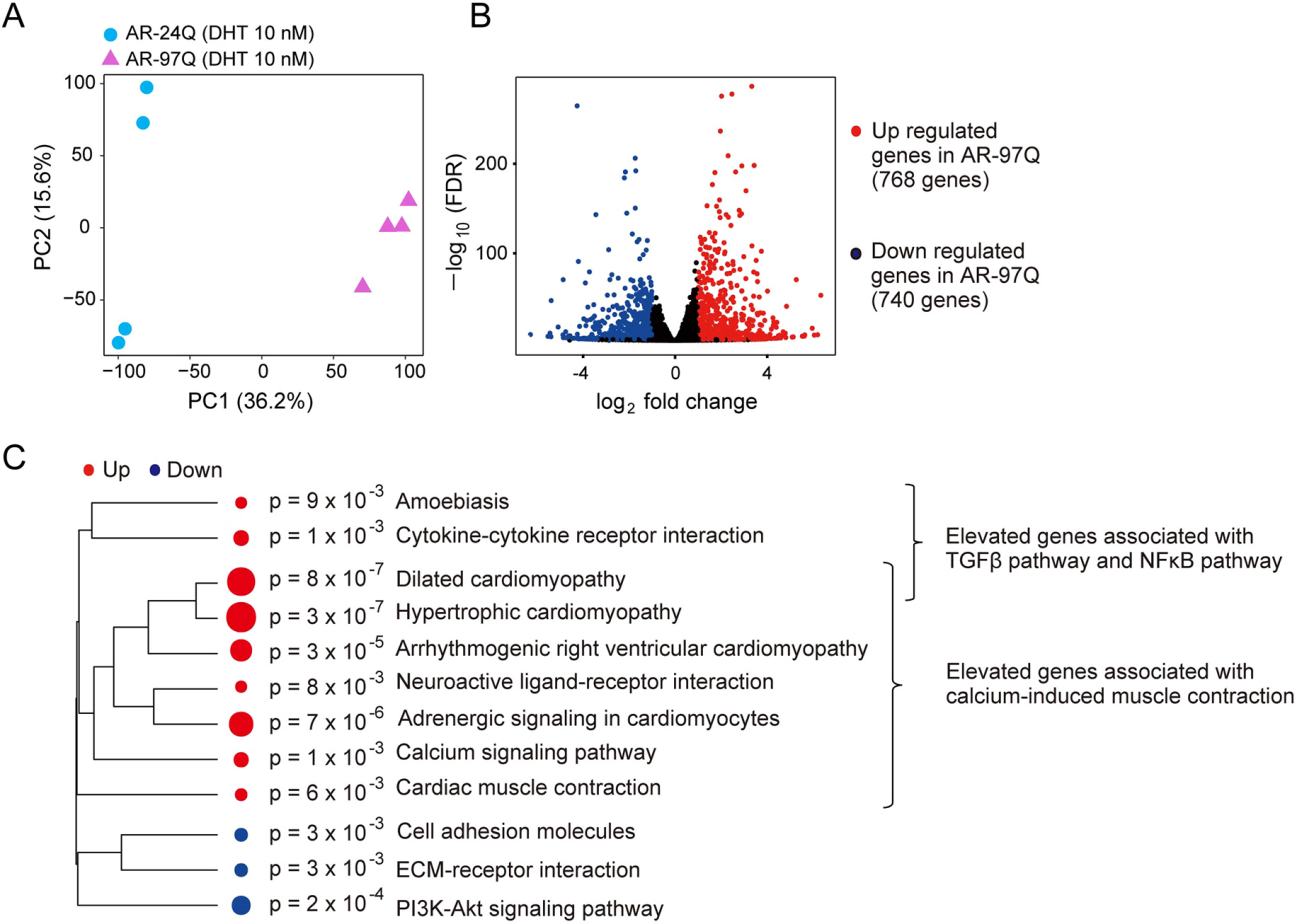
**Identification of disease-associated pathways via comparative analysis of expression profiles between AR-24Q and AR-97Q cells.** (A) PCA of total gene expression profiles. (B) Volcano plots showing the comparison of DEGs between AR-97Q and AR-24Q cells. Red dots indicate upregulated DEGs, and blue dots indicate downregulated DEGs in AR-97Q cells. (C) Representative pathways of upregulated and downregulated DEGs in AR-97Q cells identified via pathway enrichment analysis. ECM, extracellular matrix.

Next, we aimed to expand the applicability of our analysis method by identifying potential effective compounds for SBMA. There are few identified drug candidates for the treatment of muscle pathology caused by SBMA, and the mechanisms underlying dysfunction of skeletal muscles expressing pathogenic ARs remain unclear. To address this issue, we compared the transcriptional profiles of AR-24Q and AR-97Q cells to identify differentially expressed genes (DEGs) that could be potential new drug targets. In the comparative analysis of total expression profiles, differences in gene expression between AR-24Q and AR-97Q cells were observed (Fig. 3A,B). The expression of 768 genes was upregulated, and that of 740 genes was downregulated, in AR-97Q cells compared to AR-24Q cells (Tables S2 and S3). Based on the results of pathway analysis, we focused on three distinct signaling cascades that are enriched in AR-97Q cells: the transforming growth factor β (TGFβ), NFκB and calcium channel-related signaling pathways (Fig. 3C). The pathways involved in amoebiasis, cytokine‒cytokine receptor interaction and dilated cardiomyopathy involve several genes, such as *Tgfb2* and *Il1b*, which are related to TGFβ signaling and NFκB signaling (Table S4). A previous report demonstrated dysregulation of the TGFβ signaling pathway in SBMA (Katsuno et al., 2010), which is upstream of the JNK and p38 MAPK [also known as mitogen-activated protein kinase (MAPK)14] signaling pathways. The expression of genes involved in these pathways was increased in AR-97Q cells, as illustrated in the scheme of Kyoto Encyclopedia of Genes and Genomes (KEGG) pathways (Fig. S3A,B). Activation of NFκB signaling in SBMA has also been previously reported (Iida et al., 2015), and the expression of IL-1β (Lim et al., 2021), an upstream molecule of the NFκB signaling pathway, was upregulated in AR-97Q cells (Fig. S3C). Multiple genes related to calcium signaling were upregulated in AR-97Q cells, including *Cacna1s*, *Cav1* and *Dhpr* (also known as *Qdpr*) (Fig. S3D,E). CaV1.1 is a voltage sensor for excitation-contraction binding in skeletal muscle (Bannister and Beam, 2013). Calcium channel signaling appears to play an important role in the differing state of AR-97Q cells from that of AR-24Q cells, warranting further investigation. The enrichment of ‘cell adhesion molecules’, ‘ECM-receptor interaction’ and ‘PI3K-Akt signaling pathway’ in the downregulated genes was considered to indicate loss of AR-97Q cell function due to DHT stimulation. To validate the results of RNA-sequencing (RNA-seq) analysis, immunoblot analysis was conducted. The protein levels of TGFβR, JNK, NFκB p65, IL-1β and Serca2 (also known as ATP2A2), the mRNA levels of which were shown to be upregulated in AR-97Q cells in RNA-seq analysis, were increased in AR-97Q cells compared to AR-24Q cells (Fig. S4).

To target these enriched representative pathways in AR-97Q cells, we selected five compounds as potential candidates capable of modulating the disease state of SBMA: naratriptan (NRT), SB202190, BAY 11-7082, N-acetylcysteine (NAC) and nifedipine (NIF). NRT is a selective agonist of the serotonin [5-hydroxytryptamine (5-HT)]1B/D (also known as HTR1B/D) receptor, and its effect on SBMA was previously reported in both a neuronal cell model of SBMA (SH-SY5Y) and the spinal cord tissue of AR-97Q mice but not in muscular cells (Minamiyama et al., 2012). NRT suppresses the cytotoxic effect of calcitonin gene-related peptide α (CGRP1; also known as CALCA) by inducing dual-specificity protein phosphatase 1 (DUSP1) and inhibiting the JNK pathway in the central nervous system. The present study re-emphasized the importance of the TGFβ and JNK pathways in myoblasts. SB202190, a potent p38 MAPK inhibitor targeting p38α/β, has been shown to promote myotube formation in C2C12-βgeo cells (Weston et al., 2003). We selected NRT and SB202190 as potential candidates for modulating dysfunction of the TGFβ–MAPK signaling pathway in SBMA. We also selected BAY 11-7082, an inhibitor of the NFκB pathway and IL-1β secretion (Gao et al., 2022; Mori et al., 2002), and NAC, an inhibitor of the NFκB pathway (Pajonk et al., 2002), as therapeutic candidates for SBMA. NAC has been reported to suppress myotube atrophy in C2C12 cells via the TNF signaling pathway, which is closely related to the TGFβ pathway, and it has also been reported to have potential in the treatment of neurological diseases (Banaclocha, 2001). To increase calcium signaling in AR-97Q cells, we selected NIF, a dihydropyridine calcium channel blocker, as a candidate drug that potentially ameliorates the pathology of SBMA.

### Assessment of the recovery of AR-97Q cells using morphology-based phenotypic analysis

Using our morphology-based phenotypic analysis, we evaluated the effects of five selected compounds (NRT, SB202190, BAY 11-7082, NAC and NIF) that specifically target activated pathways in AR-97Q cells compared to AR-24Q cells. If a candidate compound has the potential to restore AR-97Q cells, as shown with PG, we would expect the morphology of AR-97Q cells to become similar to that of AR-24Q cells. We compared the morphological profiles using PCA and hierarchical clustering. First, we conducted western blot analyses to assess the effects of the selected compounds on their target pathways in AR-97Q cells (Fig. S5). The results demonstrated a decrease in the ratio of phosphorylated (p-)JNK to JNK in AR-97Q cells treated with 1 or 10 µM NRT. The ratio of p-p38 MAPK to p38 MAPK was significantly suppressed after treatment with 1 or 10 µM SB202190. The ratio of p-IκBα to IκBα (also known as NFKBIA) was reduced after administration of 10 µM BAY11-7082, and the ratio was also suppressed by 0.5 or 5 mM NAC. The ratio of p-CaMKII to CaMKII (also known as CAMK2) was also diminished by the addition of 1 or 10 µM NIF.

**Fig. 4.**
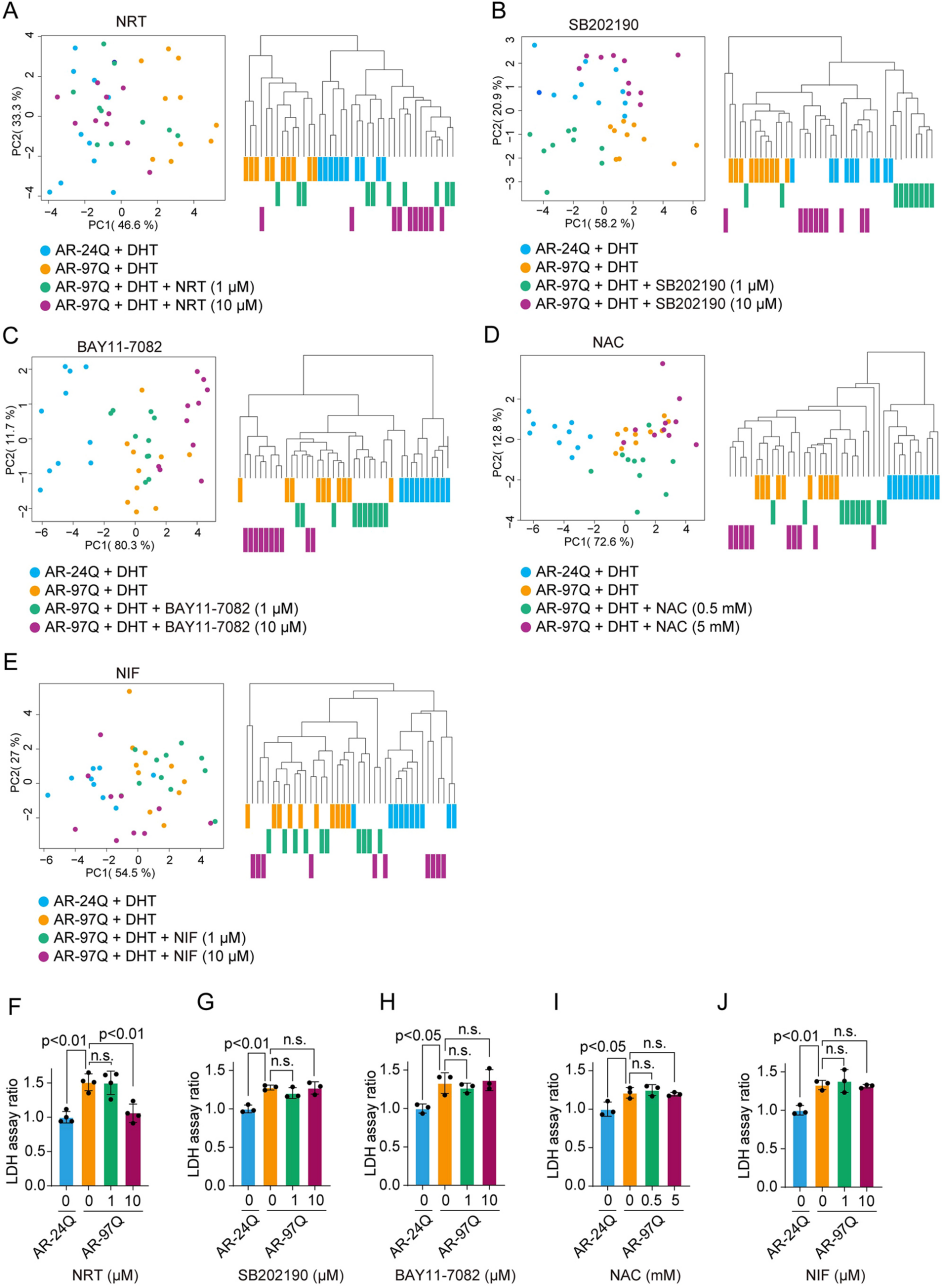
**Assessment of the phenotypic recovery of AR-97Q cells using morphology-based phenotypic analysis.** (A–E) PCA and hierarchical clustering of morphological profiles obtained from phase-contrast images of AR-24Q or AR-97Q cells that were treated with or without naratriptan (NRT) (A), SB202190 (B), BAY11-7082 (C), N-acetylcysteine (NAC) (D) and nifedipine (NIF) (E). The heatmaps of the morphological profiles are simplified, with labels corresponding to the clustering trees. (F–J) LDH release from AR-24Q and AR-97Q cells treated with or without NRT (F), SB202190 (G), BAY11-7082 (H), NAC (I) or NIF (J). Error bars represent s.d. (*N*=3). Statistical analysis was performed by one-way ANOVA with post hoc Dunnett's test. n.s., not significant.

Among the five candidate compounds, only NRT facilitated clear phenotypic recovery, shifting the morphology of AR-97Q cells towards that of AR-24Q cells at 1 µM, as shown by both PCA and hierarchical clustering (Fig. 4A). In contrast, the other four candidate compounds did not induce substantial morphological changes (Fig. 4B–E). Although SB202190 appeared to partially shift the AR-97Q morphology toward the AR-24Q phenotypes, the cluster boundaries remained ambiguous compared to the distinct effect of NRT. Furthermore, in the PCA plot (Fig. 4B), the 1 µM (green) and 10 µM (purple) SB202190-treated samples shifted in opposite directions along the PC2 axis, suggesting an inconsistent response. For these reasons, SB202190 was not retained as a lead compound. Therefore, we focused on confirming the biological changes in AR-97Q cells in response to NRT and performed an LDH assay. The recovery of NRT-treated cell morphology corresponded to a decrease in LDH levels, suggesting that our morphological profile again reflects the biological treatment effect, similar to what was revealed in the PG experiments (Fig. 2F and Fig. 4F). The other four candidate compounds did not suppress LDH levels, consistent with the findings from the morphology-based phenotypic analysis (Fig. 4G–J).

**Fig. 5.**
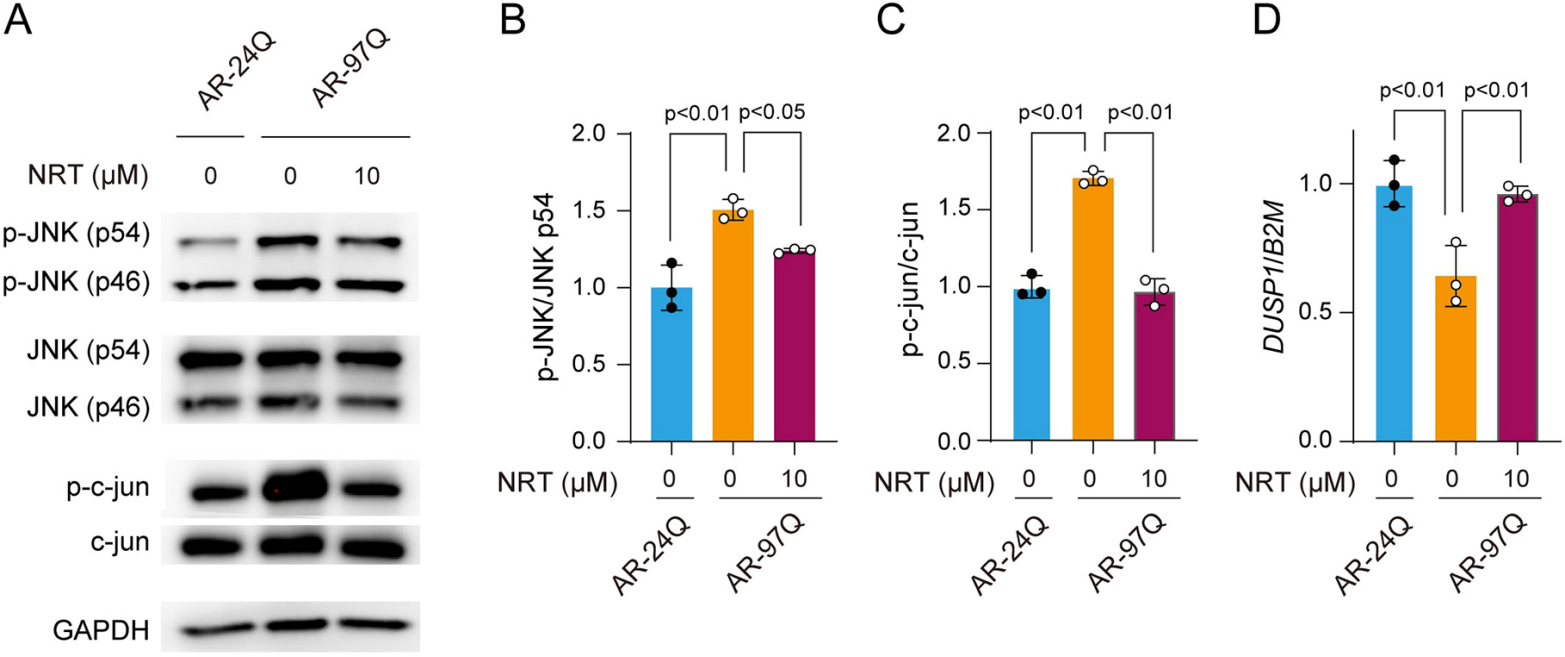
**Effect of NRT on the JNK signaling pathway in SBMA model C2C12 cells.** (A) Western blot analysis to assess the effect of NRT. Immunoblots showing the levels of phosphorylated and total forms of JNK and c-Jun proteins in AR-24Q and AR-97Q cells treated with DHT. The AR-97Q cells were treated with or without NRT. (B,C) Quantitative densitometry analysis of phosphorylated (p-)JNK and p-c-Jun levels. (D) Relative expression levels of *Dusp1*. Error bars represent s.d. (*N*=3). Statistical analysis was performed by one-way ANOVA with post hoc Dunnett's test.

In our previous study, we showed that NRT reduced cell damage in a neuronal cell model of SBMA and ameliorated polyglutamine-mediated neuronal damage in AR-97Q mice by suppressing the JNK pathway (Minamiyama et al., 2012). To assess the effects of NRT in a muscle cell model of SBMA, immunoblotting was performed to measure the levels of JNK and c-Jun (also known as JUN) (Fig. 5A–C). The levels of p-JNK and p-c-Jun were significantly greater in AR-97Q cells than in AR-24Q cells, whereas there was a significant reduction in the levels of p-JNK and p-c-Jun in AR-97Q cells following the administration of 10 µM NRT. In addition, because NRT treatment of SBMA model neuronal cells has previously been associated with the induction of DUSP1, we also measured the expression levels of *Dusp1* in the model muscle cells. As expected, NRT restored *Dusp1* transcript levels in AR-97Q cells (Fig. 5D). Although NRT targeting the 5-HT1B/D receptor has previously shown promise in alleviating the neuronal pathology of SBMA, our results suggest that inhibition of the JNK pathway could be a target for the treatment of muscle dysfunction. Furthermore, the present study demonstrated that drug candidates for the treatment of muscle pathology can be identified with our noninvasive and efficient screening method using morphology-based phenotypic analysis.

### The morphological profile can discriminate drug effects as a screening tool

Our study employed morphology-based phenotypic analysis to assess the effects of PG and NRT on SBMA model muscle cells. The analysis not only confirmed the efficacy of the potential therapeutics but also demonstrated distinct morphological responses to NRT compared to PG, as visualized by uniform manifold approximation and projection (UMAP) and PCA (Fig. S6A,B). The UMAP plot showed that the multi-parametric morphological profile of DHT-treated AR-97Q cells shifted toward that of DHT-treated AR-24Q cells, with nondisease morphology, but the trajectories differed between PG and NRT treatments. Further insight can be obtained from the PCA visualization (Fig. S6B,C), which indicated that the standard deviation of morphological parameters is greater under NRT treatment than under PG treatment. These findings suggest that NRT induces more heterogeneous morphological changes across individual cells, whereas PG leads to more uniform changes, resulting in a characteristic and consistent phenotype (Fig. S6C). This quantitative observation from the phenotypic analysis led us to hypothesize that machine learning models could be trained to differentiate these morphological responses, enabling the automated identification of drug effects on model cells.

**Fig. 6.**
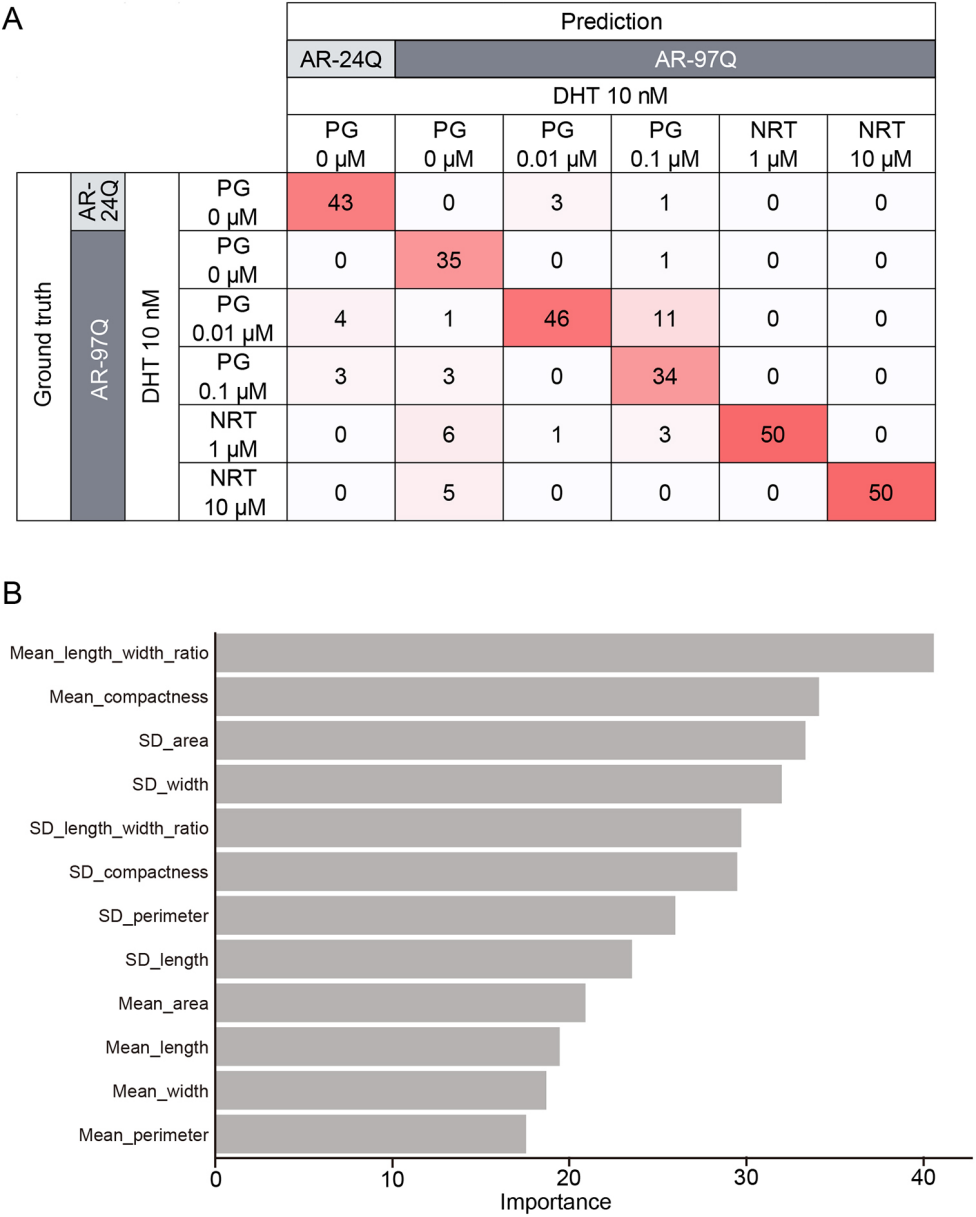
**The morphological profile can discriminate drug effects as a screening tool.** (A) Prediction performance across six drug effect categories. (B) Importance of selected morphological parameters in the drug effect category prediction model.

To test this hypothesis, we used categorical data from AR-24Q cells with no drug, AR-97Q cells with no drug, and AR-97Q cells treated with PG or NRT to train a predictive machine learning model. The model aimed to classify the drug responses (Fig. 6; Fig. S7). Our model achieved high predictive accuracy (0.86) in distinguishing between six categories of drug response. Of particular note is the exceptional discriminative performance of the model in distinguishing between drug-free cells, PG-treated cells and NRT-treated cells, which was remarkably high. These findings suggest that it is possible to distinguish between PG-type and NRT-type morphological profiles solely based on their morphological characteristics combined with our model cells (AR-24Q and AR-97Q). Consequently, this approach could be applied to develop a ‘morphology-based drug category predictor’, which would enhance drug screening processes and provide more insightful interpretative assistance.

## DISCUSSION

In the present study, we developed a noninvasive and efficient *in vitro* drug screening technique based on morphology-based phenotypic analysis. Our goal is to accelerate therapeutic advances for SBMA, with particular emphasis on addressing the muscular aspects of the disease. We used these methods to assess potential drug candidates in a neuronal cell model of SBMA (Imai et al., 2022). The mechanisms of SBMA are not fully understood, and there is a lack of effective medications for patients with SBMA. Phenotype-based approaches have great potential for assessing overall disease status and are useful for understanding pathophysiology and determining treatment efficacy. Conventional molecular biology techniques are labor intensive and sometimes too sensitive to exclude assay artifacts. These issues hinder the scalability needed to thoroughly evaluate a wide range of drug candidates and explore new approaches in drug design. Label-free phenotyping techniques have great potential to increase the efficiency and flexibility of drug effect assessment, overcoming the limitations of traditional methods. Noninvasive phenotypic assays provide objective, comprehensive and sensitive measurements of cellular responses with minimal assay artifacts. These methods also allow us to collect mass data that can provide more meaningful information for evaluating drug candidates to achieve greater efficacy and fewer side effects.

Recently, we developed a new morphological analysis concept called in silico-FOCUS using a neuronal model of SBMA generated from motor neuron-enriched embryonic mouse spinal cord cells fused to mouse neuroblastoma cells (NSC34) (Imai et al., 2022). In this method, we leveraged image-based analysis to establish an ‘image cytometry’ pipeline, enabling us to scrutinize heterogeneous subpopulations of neuronal cells. In the present study, myoblasts were cultured under confluent conditions, and we quantified their morphological profile by collecting all cells in the population. Focusing only on myotube-like cells with conventional subjective criteria (Malena et al., 2013; Milioto et al., 2017) may miss important information and not effectively track how cells respond to the drug because muscle cells from patients with SBMA are heterogeneous with hypertrophic and atrophic cells (Borgia et al., 2017). To optimize our drug screening approach that uses cell subpopulation morphologies, we used our previously established C2C12 drug effect monitoring analysis (Ishikawa et al., 2019). In brief, based on our previous findings, we collected data from all cells in the myotube differentiation images and used statistical summaries of these overall myoblast responses (Fig. 1). The effectiveness of this ‘cell population description’ approach was confirmed in the present study. This approach proved superior to the subjective selection of limited morphological parameters to define ‘myotube-like cells’ (Fig. 2B). It also allowed us not only to quantitatively distinguish between ‘nondisease’ and ‘disease’ cells but also to evaluate how the drug affected the diseased cells. It is therefore noteworthy that our ‘cell population concept’ has consistently shown robustness in morphological analysis, especially for confluent cells. Typically, label-free morphological analysis of confluent cell cultures is challenging because clear cell segmentation is difficult in such image analysis. This study demonstrated that the effective collection of data from the entire cell population, coupled with bootstrapping, results in the elucidation of robust morphological profiles of the cell population that support quantitative analyses.

In this study, the expression profiles of AR-24Q and AR-97Q muscle cells were compared, and the TGFβ, NFκB and calcium signaling pathways were found to be activated in AR-97Q cells. The JNK and p38 MAPK pathways were also activated downstream of the TGFβ pathway. Phosphorylation of c-Jun, IκBα and p38 MAPK has been reported to be intensified in the skeletal muscles of AR-97Q mice from early to advanced stages (Iida et al., 2019), which is consistent with the results of the transcriptional analysis in the present study. Phosphorylation of these molecules was also augmented in the spinal cords of AR-97Q mice at early and advanced stages (Iida et al., 2019). According to the results from microarray analysis of the spinal cords of AR-24Q and AR-97Q mice at advanced stages, members of the TGFβ and the JNK-mediated MAPK signaling pathways were upregulated in the spinal cords of AR-97Q mice, suggesting the involvement of common pathogenic pathways in the spinal cord and skeletal muscle in SBMA (Minamiyama et al., 2012). The present study showed that the level of *Dusp1* is suppressed, and that the activity of the JNK pathway is increased, in our muscle cell model of SBMA. NRT restored the *Dusp1* level and suppressed the JNK pathway. JNK is a member of the MAPK family and regulates apoptosis in response to various stresses. The JNK signaling pathway has been reported to affect not only motor neuron diseases – such as SBMA, amyotrophic lateral sclerosis and spinal muscular atrophy – but also Huntington's disease, Alzheimer's disease, schizophrenia and many other neurological disorders (Mehan et al., 2011; Perrin et al., 2009; Schellino et al., 2019; Winchester et al., 2012). Thus, the DUSP1 and JNK pathways are of interest in neuromuscular diseases other than SBMA. From this perspective, our image analysis technique can be applied to assess other neuromuscular diseases.

NIF improved muscle pathology in a mouse model of Duchenne muscular dystrophy (Altamirano et al., 2013), SB202190 promoted myotube formation in C2C12-βgeo cells (Weston et al., 2003), and NFκB inhibition improved muscle atrophy in a mouse model of cachexia (Cai et al., 2004). However, the present study did not demonstrate the efficacy of these compounds in the muscle cell SBMA model. There are several possible reasons for these discrepancies. First, the upregulated pathway in AR-97Q cells may have involved compensatory changes rather than SBMA-related pathophysiological changes. Second, the pathways we targeted may be downstream of SBMA pathogenesis, and, thus, targeting these pathways had little effect on pathogenesis.

Our discovery of distinct morphological profiles induced by PG and NRT has enabled us to develop a ‘drug effect prediction model’ based solely on these morphological characteristics. The chosen parameters for this model revealed that the unique morphological profiles associated with each drug are intricately composed of both the mean and standard deviation of these parameters, which collectively represent the morphology of the entire cell population (Fig. 6B). This finding suggests that our descriptors, which capture the heterogeneity of cell populations, offer high explanatory power in delineating subtle differences between drug effects. This approach surpasses traditional methods, such as counting myotubes, previously used for characterizing myoblast cells, in its ability to capture more nuanced information. If this methodology can be extended to a broader range of drug candidates, it has the potential to noninvasively distinguish different types of drug effects. Such a capability would greatly enhance our understanding of SBMA, especially when applied to diverse seed compound libraries.

Phenotype assays, which detect alterations in cell morphology, have gained attention for their ability to identify potential drug candidates, even when the mechanism of action is unknown (Brown and Wobst, 2020). Label-free image analysis for phenotype assays has been extensively developed, and its advantages have been discussed in previous research (King et al., 2019). Our upcoming challenge involves integrating morphology-based phenotypic analysis of myoblast cells with previously established morphology-based phenotypic analysis of motor neurons. This integration will enable us to explore more effective and lower side-effect drug candidates for SBMA. However, we still face challenges associated with daily experimental biases stemming from variations in cell lots and passages. These challenges hinder the expansion of this image-based drug screening method. To establish a more robust analysis pipeline, our next goal is to combine the technical development of stable cell lines with experimental automation. We expect that our established method will pave the way for an efficient and adaptable drug screening platform that can also be applied to other *in vitro* models of neurodegenerative diseases.

Limitations of the present study are as follows. Our morphology-based phenotypic analysis cannot be effectively used if the drug does not affect cell morphology. It is possible that there are drugs that mitigate cellular damage without morphologic changes, and such therapeutic agents may not be screened using the current morphology-based analysis. Although our data demonstrated that morphological profiles can capture subtle cellular changes that may be imperceptible to the human eye, it remains critical to biologically establish experimental systems that define thresholds for biologically meaningful morphological changes. It is challenging to distinguish meaningful information from mere noise in cell assay systems that lack appropriate positive controls. Therefore, in our upcoming endeavors, we will deepen our exploration by constructing different cell types harboring various AR mutants using receptor engineering approaches.

## MATERIALS AND METHODS

### Cells and cell culture

C2C12 mouse myoblast cells were obtained from the RIKEN Cell Bank (Tsukuba, Japan). The SBMA model cell lines AR-24Q and AR-97Q were established as previously reported (Iida et al., 2019). The cell lines were genetically modified to stably express human full-length ARs with 24 or 97 CAG repeats. They have been authenticated and tested for contamination prior to use. DHT, a natural ligand of AR, at 10 nM was used to generate disease phenotypes in AR-97Q cells. C2C12 cells were seeded at a concentration of 6.0×10^4^ cells/well in type I collagen-coated 24-well plates (AGC Techno Glass Co., Ltd., Shizuoka, Japan) in Dulbecco's modified Eagle's medium (DMEM; Wako Pure Chemical Industries, Ltd., Osaka, Japan) supplemented with 10% fetal bovine serum (Life Technologies Japan, Ltd., Tokyo, Japan) and 10 mM 4-(2-hydroxyethyl)-1-piperazineethanesulfonic acid (HEPES) buffer (Nakarai Tesque Inc., Kyoto, Japan). For cell differentiation, the medium was changed to low-glucose DMEM (Wako Pure Chemical Industries, Ltd.) supplemented with 2% horse serum (Life Technologies Japan, Ltd.). The cells were seeded on Day 0, and the medium was changed to differentiation medium on Day 1. Then, compounds and DHT were added on Day 3, and images were acquired on Day 5. The following compounds were used: PG (United States Pharmacopeial Convention, MD, USA), NRT (TCI chemicals, Tokyo, Japan), SB202190 (Chemscene, NJ, USA), BAY 11-7082 (Selleck Chemicals, TX, USA), NAC (Sigma-Aldrich, MO, USA) and NIF (Selleck Chemicals). The compounds were dissolved in dimethyl sulfoxide (DMSO) and subsequently prepared at two final concentrations: PG (0.01, 0.1 μM), NRT (1, 10 μM), SB202190 (1, 10 μM), BAY 11-7082 (1, 10 μM), NAC (0.5, 5 mM) and NIF (1, 10 μM).

### Morphology-based phenotypic analysis

On Day 5, phase-contrast microscopy images were acquired in 24-well plates using an automatic cell image acquisition system (BioStation CT, Nikon Corporation, Tokyo, Japan) at 4× magnification (a single point per well, covering 4 mm^2^; 1000 pixels^2^/image). Each image was set in the center of the well to minimize the disturbance of the meniscus and included more than 200 cells. The obtained raw images were processed to measure individual cell morphologies and obtain a summary of morphological profiles of the cell population (minimum of 200 cells to a maximum of 5000 cells collected per well). Image processing was performed by using Python version 3.7.3, with the NumPy version 1.20.0 and OpenCV version 4.4.0 packages. The image processing pipeline was designed with seven processes: (1) background adjustment, (2) enhancement of texture, (3) binarization, (4) removal of small objects, (5) closing, (6) hole filling and (7) removal of frame-touching objects (Fig. S8). From each image, six basic morphological descriptors – area, perimeter, length, width, length-to-width ratio and compactness (Table S1) – were measured per single-cell region. As described in Fig. 1, the single-cell dataset was tagged with parameters measured under the same conditions, three images from triplicate wells were pooled once, and ten bootstrap random samples (allowing overlap) were applied to obtain ten cell population samples (200 cells). For each cell population, the mean and s.d. for each morphological parameter were summarized. Therefore, 12 final morphological parameters were used for morphological profiling (Table S1). The data processing procedure was achieved by the original code using R version 4.0.2. The multicollinearity problem of the 12 morphological parameters is carefully considered, and analysis methods or models that minimize such effect on the statistical analysis have been chosen.

### Total expression analysis by RNA-seq

Total RNA was purified with an RNeasy Kit (QIAGEN, Hilden, Germany) according to the manufacturer's protocol. For total RNA, library preparation and sequencing were conducted by Nagoya University Center for Gene Research. RNA-seq was performed on a sequencer (NextSeq 550; Illumina) in accordance with the manufacturer's protocol. The raw expression data were analyzed using Illumina Dataspace applications. Gene-level expression data (read counts) were processed by using the web portal for integrated differential expression and pathway analysis (iDEP.96). iDEP.96 was used for PCA, identification of DEGs and pathway analysis. DEGs were extracted with a false discovery rate (FDR) cutoff of 0.1 and a minimum fold change of 2. Pathway enrichment analysis of DEGs was conducted with KEGG pathway analysis (Kanehisa et al., 2021; Luo and Brouwer, 2013).

### LDH assay

LDH assays were performed using a Cytotoxicity Detection Kit PLUS (Roche Diagnostics, IN, USA). Cells were cultured and differentiated in 24-well plates. The medium was extracted 48 h after treatment with the indicated concentrations of DHT, PG, NRT, SB202190, BAY11-7082, NAC and NIF. The cell culture supernatant was incubated with the substrate for 15 min at room temperature, and its absorbance was measured at 490 nm using a plate reader (Infinite M Plex, Tecan, Kanagawa, Japan).

### Immunoblotting and antibodies

The cultured cells were lysed using Cellytic MT Cell Lysis Reagent (Sigma-Aldrich) supplemented with Halt Protease and Phosphatase Inhibitor Cocktails (Thermo Fisher Scientific, MA, USA). Equal amounts of protein were loaded on 5–20% SDS‒PAGE gels (Wako, Osaka, Japan) and transferred to Hybond‒P membranes (GE Healthcare, NJ, USA). The following antibodies were used in this study: anti-p-SAPK/JNK (Thr183/Tyr185) (81E11) (4668, Cell Signaling Technology, MA, USA; 1:1000), anti-SAPK/JNK (9252, Cell Signaling Technology; 1:1000), anti-p-c-Jun (Ser63) (54B3) (2361, Cell Signaling Technology; 1:1000), anti-c-Jun (ab16777, Abcam; 1:1000), anti-GAPDH (6C5, Abcam, Cambridge, UK; 1:5000), anti-MHC (MF20, R&D Systems, MN, USA; 1:1000), anti-PPARγ (PP-A3409A-00, Perseus Proteomics, Tokyo, Japan 1:1000), anti-TGFβR (06-227, Millipore, MA, USA; 1:1000), anti-NFκB p65 (#3987, Cell Signaling Technology; 1:1000), anti-IL-1β (ab9722, Abcam; 1:1000), anti-Serca2 (4388, Cell Signaling Technology; 1:1000), anti-p-p38 MAPK (Thr180/Tyr182) (9211, Cell Signaling Technology; 1:1000), anti-p38 MAPK (9212, Cell Signaling Technology; 1:1000), anti-p-IκBα (Ser32) (sc8404, Santa Cruz Biotechnology, CA, USA; 1:1000), anti-IκBα (4814, Cell Signaling Technology; 1:1000), anti-p-CamKII (Thr286) (sc12866, Santa Cruz Biotechnology; 1:1000) and anti-CamKII (4436, Cell Signaling Technology; 1:1000). The density of each band was quantified using ImageJ software (National Institutes of Health, MD, USA).

### Quantitative RT–PCR

Total RNA was extracted from C2C12 cells using TRIzol (Invitrogen) and an RNeasy Mini Kit (QIAGEN). The extracted RNA was then reverse transcribed into first-strand cDNA using a ReverTra Ace qPCR RT Kit (Toyobo, Osaka, Japan). PCR amplification was performed using rTaq DNA polymerase (Toyobo) according to the manufacturer's protocol. Quantitative PCR was performed using THUNDERBIRD SYBR qPCR Mix (Toyobo), and the amplified products were detected with an iCycler system (Bio-Rad Laboratories, CA, USA). The expression level of the internal control, *B2m*, was simultaneously quantified. The following PCR primers were used: *B2m* forward/reverse, 5′-GTGTGAGCCAGGATATAGAAAGAC-3′/5′-AAGCCGAACATACTGAACTGC-3′; *Dusp1* forward/reverse, 5′-TGTTGTTGGATTGTCGCTCCT-3′/5′-TTGGGCACGATATGCTCCAG-3′.

### Prediction model

To predict drug effect categories, we generated training and test datasets from a single-cell data pool encompassing six different conditions: (1) AR-24Q with DHT; (2) AR-97Q with DHT; (3) AR-97Q with DHT and PG at 0.01 μM; (4) AR-97Q with DHT and PG at 0.1 μM; (5) AR-97Q with DHT and NRT at 1 μM; and (6) AR-97Q with DHT and NRT at 10 μM. For each category, datasets were created using a bootstrap method with 50 iterations, each sampling 1000 cells (allowing overlaps), as described in Fig. S7. A random forest was employed to develop a model capable of discriminating these six categories. The model utilized 12 basic morphological parameters as explanatory variables, and the six drug effect categories served as the objective parameters. Model performance was validated using a leave-category-out cross-validation approach.

### Statistical analysis

For the evaluation of morphological profile similarities and multidimensional data visualization, unsupervised statistical analysis was applied. All data visualization, PCA, hierarchical clustering (using correlation coefficients with average linkages), UMAP and machine learning (random forest) were performed using R (version 3.4.1). The performances of the machine learning models were tested via leave-and-condition-out cross-validation. We analyzed the data with an unpaired two-sided *t*-test for comparisons of two groups and one-way ANOVA with post hoc Dunnett's test for multiple comparisons unless otherwise noted. Tests were conducted with GraphPad Prism 8 (GraphPad). Significance (*P*<0.05) is indicated in each figure.

## Supplementary Material

10.1242/dmm.052220_sup1 Supplementary information

## References

[DMM052220C1] Altamirano, F., Valladares, D., Henríquez-Olguín, C., Casas, M., López, J. R., Allen, P. D. and Jaimovich, E. (2013). Nifedipine treatment reduces resting calcium concentration, oxidative and apoptotic gene expression, and improves muscle function in dystrophic mdx mice. *PLoS ONE* 8, e81222. 10.1371/journal.pone.0081222 24349043 PMC3857175

[DMM052220C2] Banaclocha, M. M. (2001). Therapeutic potential of N-acetylcysteine in age-related mitochondrial neurodegenerative diseases. *Med. Hypotheses* 56, 472-477. 10.1054/mehy.2000.1194 11339849

[DMM052220C3] Bannister, R. A. and Beam, K. G. (2013). CaV1.1: the atypical prototypical voltage-gated Ca2+channel. *Biochim. Biophys. Acta Biomembr.* 1828, 1587-1597. 10.1016/j.bbamem.2012.09.007 PMC3615030 22982493

[DMM052220C4] Borgia, D., Malena, A., Spinazzi, M., Desbats, M. A., Salviati, L., Russell, A. P., Miotto, G., Tosatto, L., Pegoraro, E., Sorarú, G. et al. (2017). Increased mitophagy in the skeletal muscle of spinal and bulbar muscular atrophy patients. *Hum. Mol. Genet.* 26, 1087-1103. 10.1093/hmg/ddx019 28087734 PMC5409076

[DMM052220C5] Brown, D. G. and Wobst, H. J. (2020). Opportunities and challenges in phenotypic screening for neurodegenerative disease research. *J. Med. Chem.* 63, 1823-1840. 10.1021/acs.jmedchem.9b00797 31268707

[DMM052220C6] Cai, D., Daniel Frantz, J., Tawa, N. E., Melendez, P. A., Oh, B.-C., Lidov, H. G. W., Hasselgren, P.-O., Frontera, W. R., Lee, J., Glass, D. J. et al. (2004). IKKbeta/NF-kappaB activation causes severe muscle wasting in mice. *Cell* 119, 285-298. 10.1016/j.cell.2004.09.027 15479644

[DMM052220C7] Cortes, C. J., Ling, S. C., Guo, L. T., Hung, G., Tsunemi, T., Ly, L., Tokunaga, S., Lopez, E., Sopher, B. L., Bennett, C. F. et al. (2014). Muscle expression of mutant androgen receptor accounts for systemic and motor neuron disease phenotypes in spinal and bulbar muscular atrophy. *Neuron* 82, 295-307. 10.1016/j.neuron.2014.03.001 24742458 PMC4096235

[DMM052220C8] D'Emilio, A., Biagiotti, L., Burattini, S., Battistelli, M., Canonico, B., Evangelisti, C., Ferri, P., Papa, S., Martelli, A. M. and Falcieri, E. (2010). Morphological and biochemical patterns in skeletal muscle apoptosis. *Histol. Histopathol.* 25, 21-32. 10.14670/HH-25.21 19924638

[DMM052220C9] Fujitani, M., Huddin, N. S., Kawai, S., Kanie, K., Kiyota, Y., Shimizu, K., Honda, H. and Kato, R. (2017). Morphology-based non-invasive quantitative prediction of the differentiation status of neural stem cells. *J. Biosci. Bioeng.* 124, 351-358. 10.1016/j.jbiosc.2017.04.006 28465021

[DMM052220C10] Gao, Q., Yang, Y., Feng, Y., Quan, W., Luo, Y., Wang, H., Zheng, J., Chen, X., Huang, Z., Chen, X. et al. (2022). Effects of the NF-κB signaling pathway inhibitor BAY11-7082 in the replication of ASFV. *Viruses* 14, 297. 10.3390/v14020297 35215890 PMC8877168

[DMM052220C11] Häcker, G. (2000). The morphology of apoptosis. *Cell Tissue Res.* 301, 5-17. 10.1007/s004410000193 10928277

[DMM052220C12] Hijikata, Y., Katsuno, M., Suzuki, K., Hashizume, A., Araki, A., Yamada, S., Inagaki, T., Iida, M., Noda, S., Nakanishi, H. et al. (2016). Impaired muscle uptake of creatine in spinal and bulbar muscular atrophy. *Ann. Clin. Transl. Neurol.* 3, 537-546. 10.1002/acn3.324 27386502 PMC4931718

[DMM052220C13] Iida, M., Katsuno, M., Nakatsuji, H., Adachi, H., Kondo, N., Miyazaki, Y., Tohnai, G., Ikenaka, K., Watanabe, H., Yamamoto, M. et al. (2015). Pioglitazone suppresses neuronal and muscular degeneration caused by polyglutamine-expanded androgen receptors. *Hum. Mol. Genet.* 24, 314-329. 10.1093/hmg/ddu445 25168383

[DMM052220C14] Iida, M., Sahashi, K., Kondo, N., Nakatsuji, H., Tohnai, G., Tsutsumi, Y., Noda, S., Murakami, A., Onodera, K., Okada, Y. et al. (2019). Src inhibition attenuates polyglutamine-mediated neuromuscular degeneration in spinal and bulbar muscular atrophy. *Nat. Commun.* 10, 4262. 10.1038/s41467-019-12282-7 31537808 PMC6753158

[DMM052220C15] Imai, Y., Yoshida, K., Matsumoto, M., Okada, M., Kanie, K., Shimizu, K., Honda, H. and Kato, R. (2018). In-process evaluation of culture errors using morphology-based image analysis. *Regen. Ther.* 9, 15-23. 10.1016/j.reth.2018.06.001 30525071 PMC6222266

[DMM052220C16] Imai, Y., Iida, M., Kanie, K., Katsuno, M. and Kato, R. (2022). Label-free morphological sub-population cytometry for sensitive phenotypic screening of heterogenous neural disease model cells. *Sci. Rep.* 12, 9296. 10.1038/s41598-022-12250-0 35710681 PMC9203459

[DMM052220C17] Ishikawa, K., Yoshida, K., Kanie, K., Omori, K. and Kato, R. (2019). Morphology-based analysis of myoblasts for prediction of myotube formation. *SLAS Discov.* 24, 47-56. 10.1177/2472555218793374 30102873

[DMM052220C18] Kanehisa, M., Furumichi, M., Sato, Y., Ishiguro-Watanabe, M. and Tanabe, M. (2021). KEGG: Integrating viruses and cellular organisms. *Nucleic Acids Res.* 49, D545-D551. 10.1093/nar/gkaa970 33125081 PMC7779016

[DMM052220C19] Kato, R., Matsumoto, M., Sasaki, H., Joto, R., Okada, M., Ikeda, Y., Kanie, K., Suga, M., Kinehara, M., Yanagihara, K. et al. (2016). Parametric analysis of colony morphology of non-labelled live human pluripotent stem cells for cell quality control. *Sci. Rep.* 6, 34009. 10.1038/srep34009 27667091 PMC5036041

[DMM052220C20] Katsuno, M., Adachi, H., Minamiyama, M., Waza, M., Doi, H., Kondo, N., Mizoguchi, H., Nitta, A., Yamada, K., Banno, H. et al. (2010). Disrupted transforming growth factor-β signaling in spinal and bulbar muscular atrophy. *J. Neurosci.* 30, 5702-5712. 10.1523/JNEUROSCI.0388-10.2010 20410122 PMC6632356

[DMM052220C21] Katsuno, M., Tanaka, F., Adachi, H., Banno, H., Suzuki, K., Watanabe, H. and Sobue, G. (2012). Pathogenesis and therapy of spinal and bulbar muscular atrophy (SBMA). *Prog. Neurobiol.* 99, 246-256. 10.1016/j.pneurobio.2012.05.007 22609045

[DMM052220C22] Kawai, S., Sasaki, H., Okada, N., Kanie, K., Yokoshima, S., Fukuyama, T., Honda, H. and Kato, R. (2016). Morphological evaluation of nonlabeled cells to detect stimulation of nerve growth factor expression by Lyconadin B. *J. Biomol. Screen.* 21, 795-803. 10.1177/1087057116645500 27126164

[DMM052220C23] Kennedy, W. R., Alter, M. and Sung, J. H. (1968). Progressive proximal spinal and bulbar muscular atrophy of late onset. *Neurology* 18, 671-680. 10.1212/WNL.18.7.671 4233749

[DMM052220C24] King, D., Glynn, M. D., Cindric, S., Kernan, D., O'Connell, T., Hakimjavadi, R., Kearney, S., Ackermann, T., Berbel, X. M., Llobera, A. et al. (2019). Label-free multi parameter optical interrogation of endothelial activation in single cells using a lab on a disc platform. *Sci. Rep.* 9, 4157. 10.1038/s41598-019-40612-8 30858536 PMC6411894

[DMM052220C25] Lim, W. F., Forouhan, M., Roberts, T. C., Dabney, J., Ellerington, R., Speciale, A. A., Manzano, R., Lieto, M., Sangha, G., Banerjee, S. et al. (2021). Gene therapy with AR isoform 2 rescues spinal and bulbar muscular atrophy phenotype by modulating AR transcriptional activity. *Sci. Adv.* 7, eabi6896. 10.1126/sciadv.abi6896 34417184 PMC8378820

[DMM052220C26] Luo, W. and Brouwer, C. (2013). Pathview: an R/Bioconductor package for pathway-based data integration and visualization. *Bioinformatics* 29, 1830-1831. 10.1093/bioinformatics/btt285 23740750 PMC3702256

[DMM052220C27] Malena, A., Pennuto, M., Tezze, C., Querin, G., D'Ascenzo, C., Silani, V., Cenacchi, G., Scaramozza, A., Romito, S., Morandi, L. et al. (2013). Androgen-dependent impairment of myogenesis in spinal and bulbar muscular atrophy. *Acta Neuropathol.* 126, 109-121. 10.1007/s00401-013-1122-9 23644820

[DMM052220C28] Mehan, S., Meena, H., Sharma, D. and Sankhla, R. (2011). JNK: a stress-activated protein kinase therapeutic strategies and involvement in Alzheimer's and various neurodegenerative abnormalities. *J. Mol. Neurosci.* 43, 376-390. 10.1007/s12031-010-9454-6 20878262

[DMM052220C29] Milioto, C., Malena, A., Maino, E., Polanco, M. J., Marchioretti, C., Borgia, D., Gomes Pereira, M., Blaauw, B., Lieberman, A. P., Venturini, R. et al. (2017). Beta-agonist stimulation ameliorates the phenotype of spinal and bulbar muscular atrophy mice and patient-derived myotubes. *Sci. Rep.* 7, 41046. 10.1038/srep41046 28117338 PMC5259768

[DMM052220C30] Minamiyama, M., Katsuno, M., Adachi, H., Doi, H., Kondo, N., Iida, M., Ishigaki, S., Fujioka, Y., Matsumoto, S., Miyazaki, Y. et al. (2012). Naratriptan mitigates CGRP1-associated motor neuron degeneration caused by an expanded polyglutamine repeat tract. *Nat. Med.* 18, 1531-1538. 10.1038/nm.2932 23023499

[DMM052220C31] Mori, N., Yamada, Y., Ikeda, S., Yamasaki, Y., Tsukasaki, K., Tanaka, Y., Tomonaga, M., Yamamoto, N. and Fujii, M. (2002). Bay 11-7082 inhibits transcription factor NF-κB and induces apoptosis of HTVL-I-infected T-cell lines and primary adult T-cell leukemia cells. *Blood* 100, 1828-1834. 10.1182/blood-2002-01-0151 12176906

[DMM052220C32] Pajonk, F., Riess, K., Sommer, A. and McBride, W. H. (2002). N-acetyl-L-cysteine inhibits 26S proteasome function: implications for effects on NF-κB activation. *Free Radic. Biol. Med.* 32, 536-543. 10.1016/S0891-5849(02)00743-8 11958954

[DMM052220C33] Perrin, V., Dufour, N., Raoul, C., Hassig, R., Brouillet, E., Aebischer, P., Luthi-Carter, R. and Déglon, N. (2009). Implication of the JNK pathway in a rat model of Huntington's disease. *Exp. Neurol.* 215, 191-200. 10.1016/j.expneurol.2008.10.008 19022249

[DMM052220C34] Sasaki, H., Takeuchi, I., Okada, M., Sawada, R., Kanie, K., Kiyota, Y., Honda, H. and Kato, R. (2014). Label-free morphology-based prediction of multiple differentiation potentials of human mesenchymal stem cells for early evaluation of intact cells. *PLoS ONE* 9, e93952. 10.1371/journal.pone.0093952 24705458 PMC3976343

[DMM052220C35] Sasaki, K., Sasaki, H., Takahashi, A., Kang, S., Yuasa, T. and Kato, R. (2016). Non-invasive quality evaluation of confluent cells by image-based orientation heterogeneity analysis. *J. Biosci. Bioeng.* 121, 227-234. 10.1016/j.jbiosc.2015.06.012 26183859

[DMM052220C36] Schellino, R., Boido, M. and Vercelli, A. (2019). JNK signaling pathway involvement in spinal cord neuron development and death. *Cells* 8, 1576. 10.3390/cells8121576 31817379 PMC6953032

[DMM052220C37] Sobue, G., Hashizume, Y., Mukai, E., Hirayama, M., Mitsuma, T. and Takahashi, A. (1989). X-linked recessive bulbospinal neuronopathy a clinicopathological study. *Brain* 112, 209-232. 10.1093/brain/112.1.209 2917278

[DMM052220C38] Sorarù, G., D'Ascenzo, C., Polo, A., Palmieri, A., Baggio, L., Vergani, L., Gellera, C., Moretto, G., Pegoraro, E. and Angelini, C. (2008). Spinal and bulbar muscular atrophy: Skeletal muscle pathology in male patients and heterozygous females. *J. Neurol. Sci.* 264, 100-105. 10.1016/j.jns.2007.08.012 17854832

[DMM052220C39] Takemoto, Y., Imai, Y., Kanie, K. and Kato, R. (2021). Predicting quality decay in continuously passaged mesenchymal stem cells by detecting morphological anomalies. *J. Biosci. Bioeng.* 131, 198-206. 10.1016/j.jbiosc.2020.09.022 33121889

[DMM052220C40] Weston, A. D., Sampaio, A. V., Ridgeway, A. G. and Underhill, T. M. (2003). Inhibition of p38 MAPK signaling promotes late stages of myogenesis. *J. Cell Sci.* 116, 2885-2893. 10.1242/jcs.00525 12771182

[DMM052220C41] Winchester, C. L., Ohzeki, H., Vouyiouklis, D. A., Thompson, R., Penninger, J. M., Yamagami, K., Norrie, J. D., Hunter, R., Pratt, J. A. and Morris, B. J. (2012). Converging evidence that sequence variations in the novel candidate gene MAP2K7 (MKK7) are functionally associated with schizophrenia. *Hum. Mol. Genet.* 21, 4910-4921. 10.1093/hmg/dds331 22899651

[DMM052220C42] Yu, Z., Dadgar, N., Albertelli, M., Gruis, K., Jordan, C., Robins, D. M. and Lieberman, A. P. (2006). Androgen-dependent pathology demonstrates myopathic contribution to the Kennedy disease phenotype in a mouse knock-in model. *J. Clin. Investig.* 116, 2663-2672. 10.1172/JCI28773 16981011 PMC1564432

