# Supplementary material for: Label-free morphology-based phenotypic analysis of spinal and bulbar muscular atrophy muscle cell models
Source: Dis Model Mech. 2025 Jun 25;18(6):dmm052220. doi: 10.1242/dmm.052220 (PMC12233066; doi:10.1242/dmm.052220)
Supplement: Supplementary information [file dmm-18-052220-s1.pdf]

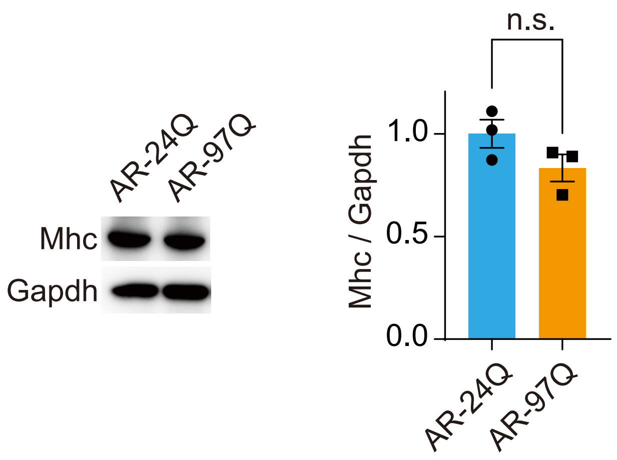

Fig. S1. Differentiation levels of AR-24Q and AR-97Q cells.

Immunoblots and quantitative densitometry analysis showing the levels of Mhc in AR-24Q and AR-97Q cells differentiated for 48 hours. AR-24Q and AR-97Q cells were treated with DHT. Error bars indicate the SEM (N = 3). Statistical analysis was performed by unpaired two-sided t tests. n.s., not significant.

Disease Models & Mechanisms • Supplementary information

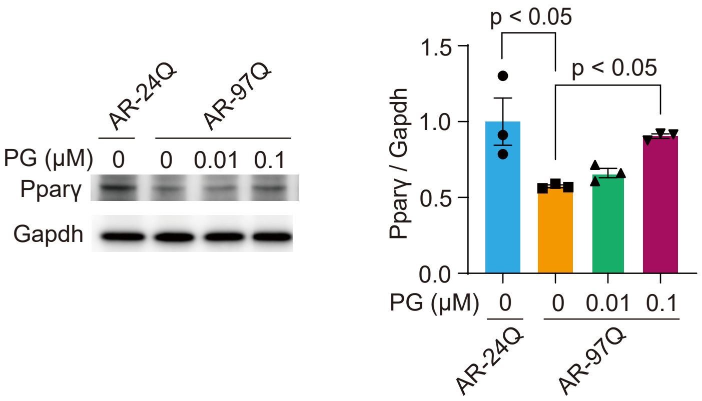

Fig. S2. The protein levels of Pparγ in AR-24Q and AR-97Q cells treated with or without pioglitazone (PG).

Immunoblots and quantitative densitometry analysis showing the levels of Pparγ in AR-24Q and AR-97Q cells. The cells were treated with DHT, and AR-97Q cells were treated with or without 0.01 or 0.1μM of PG. Error bars indicate the SEM (N = 3). Statistical analysis was performed by one-way ANOVA with post hoc Dunnett's test.

Disease Models & Mechanisms • Supplementary information

A TGFβ signaling pathway

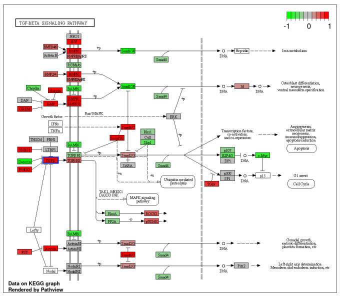

B MAPK signaling pathway

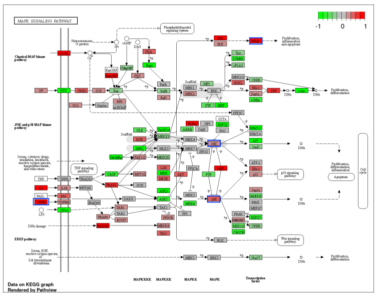

C NFκB signaling pathway

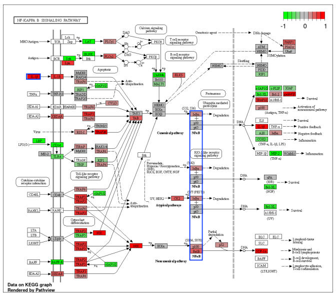

D Calcium signaling pathway

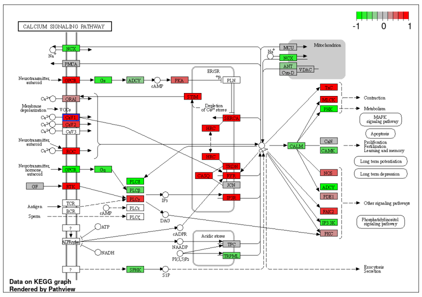

E Adrenergic signaling pathway

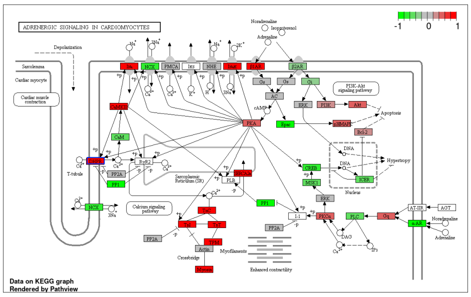

Fig. S3. Significantly enriched pathways identified via KEGG pathway enrichment analysis of DEGs

A–E. The upregulated genes in AR-97Q cells are colored red, and the downregulated genes in AR-97Q cells are colored green for the TGFβ signaling pathway (**A**), the MAPK signaling pathway (**B**), the NFκB pathway (**C**), the calcium signaling pathway (**D**), and the adrenergic signaling pathway (**E**). The cutoff criteria were set at an FDR < 0.1 and a fold change (log2) < 2. The areas with blue box indicate molecules of interest.

Disease Models & Mechanisms • Supplementary information

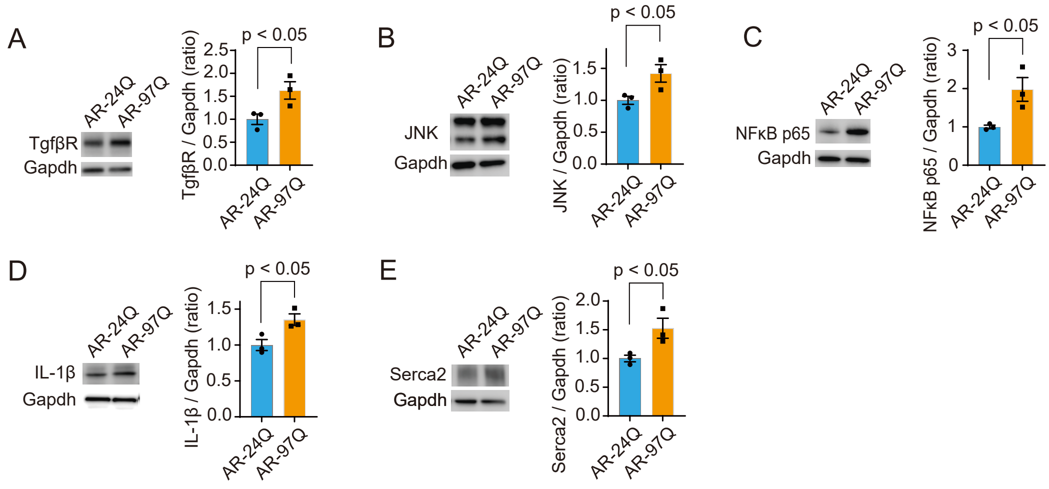

Fig. S4. Validation of gene expression analysis of AR-24Q and AR-97Q cells.

A–**E.** Immunoblots and quantitative densitometry analysis showing the levels of TgfβR (**A**), JNK (**B**), NFκB p65 (**C**), IL-1β (**D**), and Serca2 (**E**) in AR-24Q and AR-97Q cells treated with DHT. The error bars represent the SEM (N = 3). Statistical analysis was performed by one-way ANOVA with post hoc Dunnett's test.

Disease Models & Mechanisms • Supplementary information

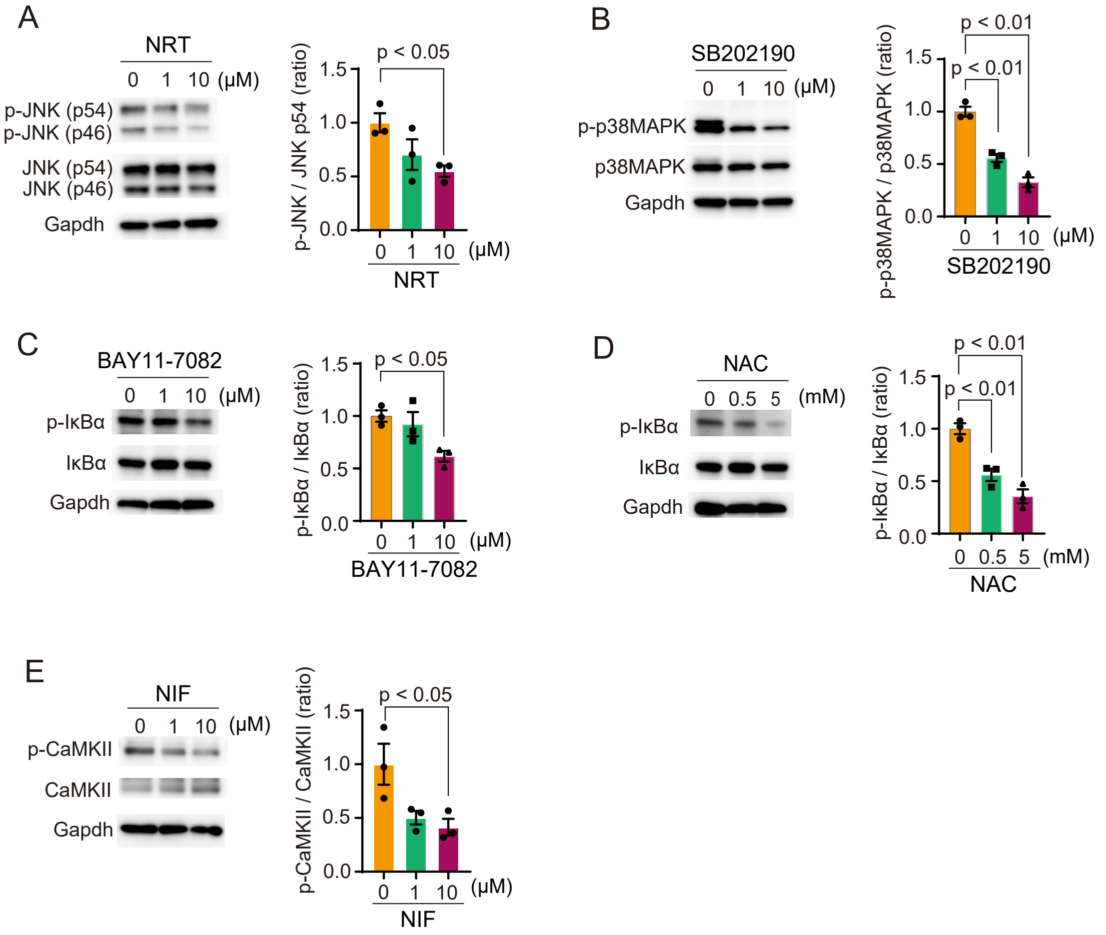

Fig. S5. The effects of the selected drugs on their target pathways.

A. Immunoblots and quantitative densitometry analysis showing the levels of phosphorylated JNK (p-JNK) in AR-97Q cells treated with NRT. **B.** Immunoblots and quantitative densitometry analysis showing the levels of phosphorylated p38MAPK (p-p38MAPK) in AR-97Q cells treated with SB202190. **C.** Immunoblots and quantitative densitometry analysis showing the levels of phosphorylated IκBα (p-IκBα) in AR-97Q cells treated with BAY11-7082. **D.** Immunoblots and quantitative densitometry analysis showing the levels of phosphorylated IκBα (p-IκBα) in AR-97Q cells treated with NAC. **E.** Immunoblots and quantitative densitometry analysis showing the levels of phosphorylated CaMKII (p-CaMKII) in AR-97Q cells treated with NIF. AR-97Q cells were treated with DHT. The error bars represent the SEM (N = 3). Statistical analysis was performed by one-way ANOVA with post hoc Dunnett's test.

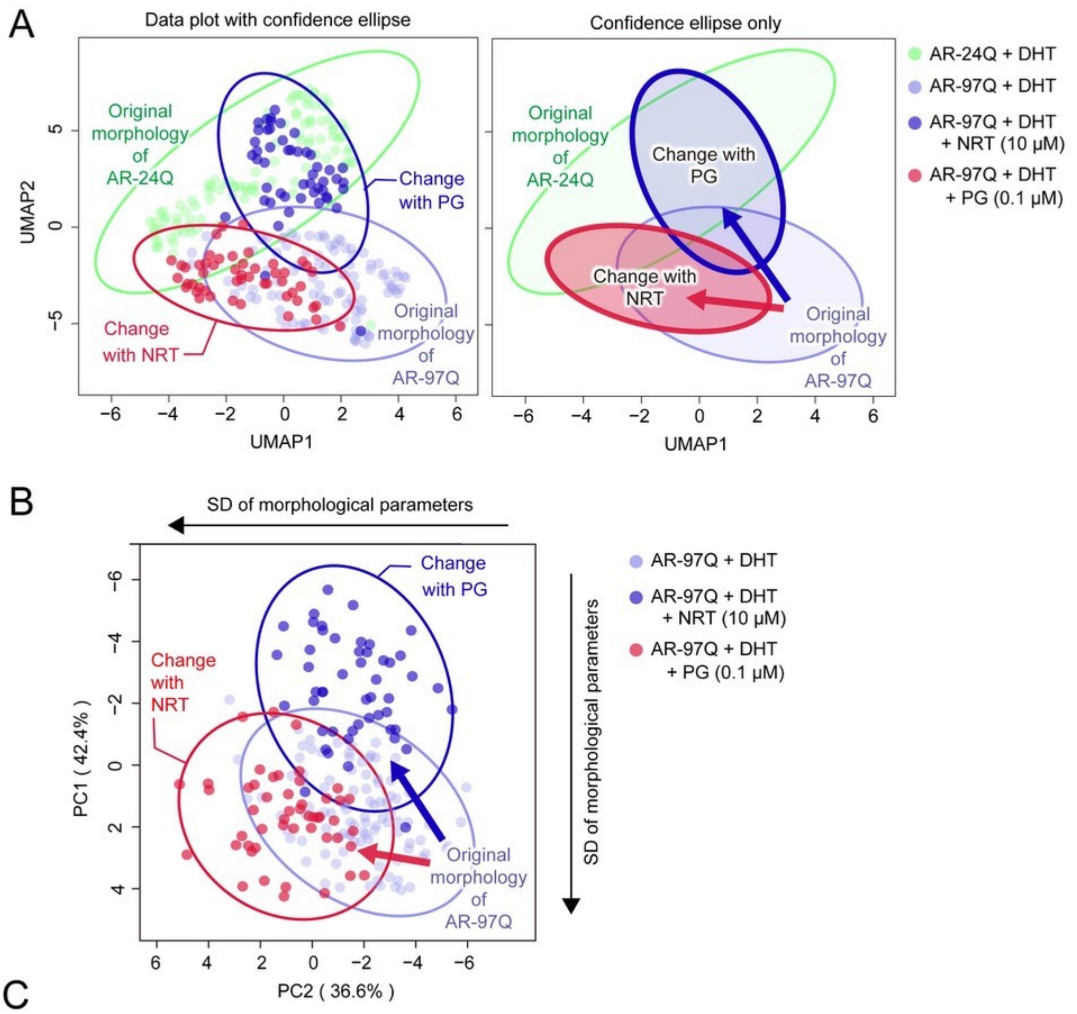

C

Morphological parameter	PC1
SD_perimeter	0.42
SD_compactness	0.40
SD_area	0.40
SD_width	0.39
SD_length	0.37
Mean_compactness	0.24
Mean_perimeter	0.21
Mean_area	0.15
Mean_width	0.14
Mean_length	0.11
Mean_length_width_ratio	-0.15
SD_length_width_ratio	-0.22

Morphological parameter	PC2
SD_length	0.22
SD_area	0.20
SD_width	0.19
SD_perimeter	0.16
SD_compactness	0.06
Mean_length_width_ratio	-0.02
SD_length_width_ratio	-0.03
Mean_compactness	-0.32
Mean_perimeter	-0.42
Mean_area	-0.42
Mean_width	-0.44
Mean_length	-0.45

Fig. S6. Differences in morphology induced by NRT or PG in AR-97Q cells.

A. Uniform Manifold Approximation and Projection (UMAP) visualization of morphological profiles of AR-24Q (treated with DHT) and AR-97Q (treated with DHT) cells under untreated, naratriptan (NRT), or pioglitazone (PG) conditions (UMAP parameters: *n_neighbors* = 15, *min_dist* = 0.5). The left panel shows individual data points overlaid with 95% confidence ellipses, while the right panel shows only confidence ellipses to emphasize the directional

changes in morphological profiles induced by PG or NRT treatment. **B.** Principal component analysis (PCA) visualization of AR-97Q cells (treated with DHT), showing the morphological shift caused by NRT or PG treatment. Confidence ellipses illustrate the separation between the two treatment conditions, highlighting the distinct directions of morphological change. **C.** Morphological features contributing to PC1 and PC2 in panel B. Features related to the standard deviation of morphological parameters, reflecting morphological diversity, contributed more to PC1 and were elevated in NRT-treated cells compared to PG-treated cells.

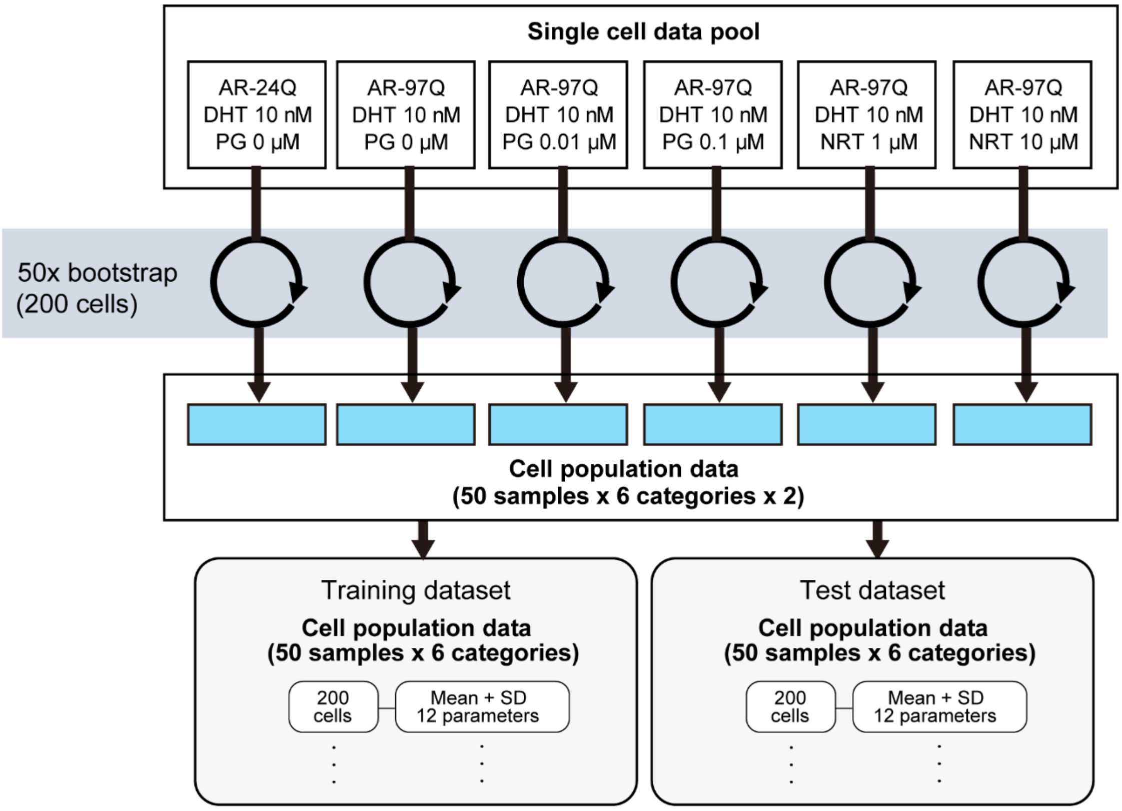

Fig. S7. The pipeline for creating the training and test dataset for the drug effect prediction model.

Disease Models & Mechanisms • Supplementary information

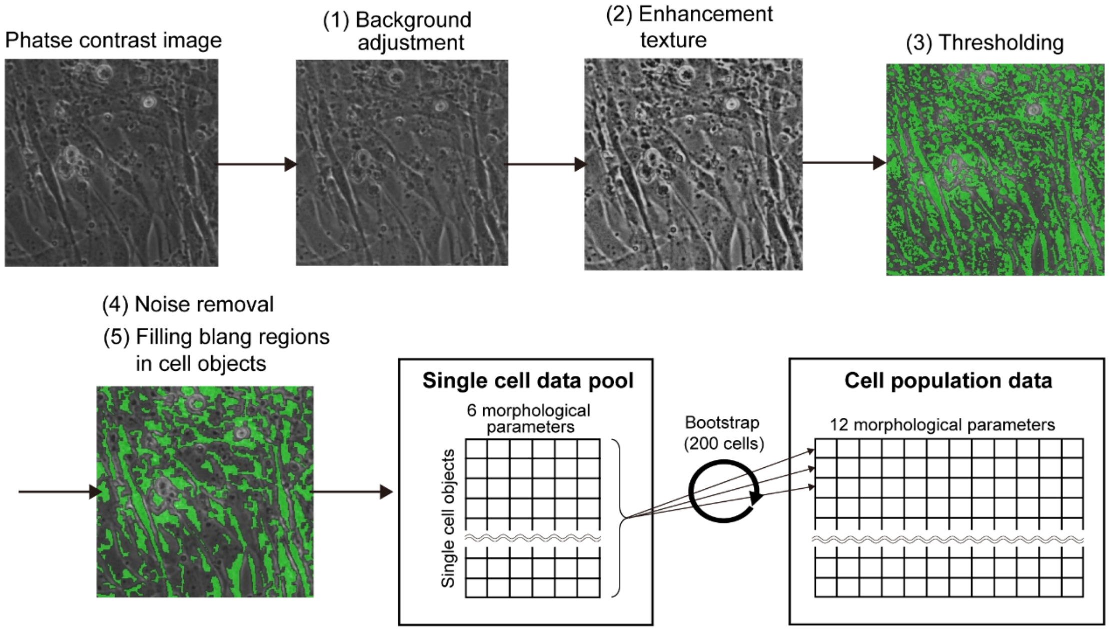

Fig. S8. Schematic diagram of the image processing pipeline.

Table S1. Morphological parameters used in the morphology-based phenotypic analysis

Parameter number	Statistics	Parameter name	Definition
1	Mean	Mean_area	Total pixels in the recognized cell region.
2		Mean_perimeter	The arc length of recognized cell region.
3		Mean_length	Long axis of bounding rectangle covering cell.
4		Mean_width	Short axis of bounding rectangle covering cell.
5		Mean_length_width_ratio	Length/Width
6		Mean_compactness	(Perimeter)^2/Area
7	Standard deviation (SD)	SD_area	Total pixels in the recognized cell region.
8		SD_perimeter	The arc length of recognized cell region.
9		SD_length	Long axis of bounding rectangle covering cell.
10		SD_width	Short axis of bounding rectangle covering cell.
11		SD_length_width_ratio	Length/Width
12		SD_compactness	(Perimeter)^2/Area

Table S2. Top 20 genes upregulated in AR-97Q cells compared to AR-24Q cells

Gene Symbol	Ensembl ID	log2 Fold Change*	Adj. p-val
**Gm6213**	ENSMUSG00000109827	7.06	4.29E-10
**Cd24a**	ENSMUSG00000047139	6.90	1.64E-09
**Grem2**	ENSMUSG00000050069	6.57	2.82E-08
**Tmem130**	ENSMUSG00000043388	6.37	1.23E-07
**Rbm24**	ENSMUSG00000038132	6.36	2.09E-51
**Itih1**	ENSMUSG00000006529	6.26	1.06E-07
**Gm6831**	ENSMUSG00000110639	5.99	1.80E-14
**Lrtm1**	ENSMUSG00000045776	5.94	3.41E-07
**Gm49808**	ENSMUSG00000117110	5.80	8.47E-07
**Ces2c**	ENSMUSG00000061825	5.69	6.81E-06
**Egflam**	ENSMUSG00000042961	5.68	4.13E-06
**Serpinb9e**	ENSMUSG00000062342	5.66	5.70E-07
**Lrrc2**	ENSMUSG00000032495	5.47	1.55E-05
**Zfp521**	ENSMUSG00000024420	5.37	8.04E-06
**Il23r**	ENSMUSG00000049093	5.33	2.99E-05
**Shisa2**	ENSMUSG00000044461	5.30	1.81E-05
**Sfrp2**	ENSMUSG00000027996	5.30	1.73E-69
**Lama1**	ENSMUSG00000032796	5.27	1.31E-05
**Gm5479**	ENSMUSG00000044697	4.94	2.51E-04
**Igha**	ENSMUSG00000095079	4.88	8.81E-05

* The log2 fold change is calculated as log2(AR-24Q) - log2(AR-97Q).

Disease Models & Mechanisms • Supplementary information

Table S3. Top 20 genes downregulated in AR-97Q cells compared to AR-24Q cells

Gene Symbol	Ensembl ID	log2 Fold Change*	Adj. p-val
**Cyp3a44**	ENSMUSG00000054417	-6.65	7.43E-09
**Xlr4a**	ENSMUSG00000079845	-5.98	2.15E-07
**Cyp3a41a**	ENSMUSG00000075551	-5.66	6.26E-06
**Ceacam10**	ENSMUSG00000054169	-5.41	5.90E-09
**Rtl1**	ENSMUSG00000085925	-5.36	1.81E-46
**Olfr330**	ENSMUSG00000050818	-5.21	1.91E-05
**Fa2h**	ENSMUSG00000033579	-5.07	4.59E-05
**Slurp1**	ENSMUSG00000022596	-5.04	4.32E-16
**Olfr224**	ENSMUSG00000059279	-4.83	4.63E-07
**Vldlr**	ENSMUSG00000024924	-4.83	8.37E-70
**Gramd1c**	ENSMUSG00000036292	-4.72	1.33E-10
**Klk7**	ENSMUSG00000030713	-4.68	1.33E-03
**Gm13932**	ENSMUSG00000083915	-4.65	7.26E-04
**Rian**	ENSMUSG00000097451	-4.62	1.77E-04
**Greb1**	ENSMUSG00000036523	-4.58	6.79E-06
**Hunk**	ENSMUSG00000053414	-4.57	1.47E-03
**Slitrk6**	ENSMUSG00000045871	-4.54	5.44E-04
**Odf3l2**	ENSMUSG00000035963	-4.53	5.09E-06
**Mc4r**	ENSMUSG00000047259	-4.48	2.06E-03
**5033406O09Rik**	ENSMUSG00000113769	-4.45	6.76E-04

* The log2 fold change is calculated as log2(AR-24Q) - log2(AR-97Q).

Disease Models & Mechanisms • Supplementary information

Table S4. Representative pathways of the DEGs identified by KEGG pathway enrichment analysis

Pathways	Adjusted p value	Number of genes	Upregulated genes
Amoebiasis	9.06E-03	12	Serpinb9c, Lamb3, Il1b, Il12a, Cd1d1, Col4a6, Lama1, Tgfb2, Serpinb6b, Serpinb9d, Serpinb9g, Serpinb9e
Cytokine–cytokine receptor interaction	1.11E-03	18	Il27ra, Amhr2, Il1rl1, Il1b, Il12a, Cntfr, Il21r, Il2rg, Ccl17, Inha, Gdf5, Tgfb2, Inhba, Ctf1, Il23r, Tnfsf15, Tnfrsf19, Il2rb
Dilated cardiomyopathy	8.03E-07	18	Sgca, Myl2, Atp2a3, Cacnb1, Itga7, Cacna1s, Tnnt2, Itgb6, Itga11, Tpm1, Lama1, Adrb1, Sgcg, Tgfb2, Dmd, Ttn, Cacng7, Tnnc1
Hypertrophic cardiomyopathy	3.02E-07	19	Sgca, Prkag3, Myl2, Atp2a3, Cacnb1, Edn1, Itga7, Cacna1s, Tnnt2, Itgb6, Itga11, Tpm1, Lama1, Sgcg, Tgfb2, Dmd, Ttn, Cacng7, Tnnc1
Arrhythmogenic right ventricular cardiomyopathy	2.84E-05	14	Sgca, Actn3, Atp2a3, Cacnb1, Cdh2, Itga7, Cacna1s, Itgb6, Lef1, Itga11, Lama1, Sgcg, Dmd, Cacng7
Adrenergic signaling in cardiomyocytes	8.37E-03	15	Myl2, Atp2a3, Cacnb1, Camk2a, Cacna1s, Tnnt2, Atp1b1, Creb3l1, Tpm1, Scn5a, Atp1a1, Adrb1, Myl4, Cacng7, Tnnc1
Cardiac muscle contraction	6.56E-06	16	Myl2, Trdn, Atp2a3, Cacnb1, Cox8b, Cacna1s, Tnnt2, Atp1b1, Casq2, Cox6a2, Tpm1, Atp1a1, Hrc, Myl4, Cacng7, Tnnc1
Calcium signaling pathway	1.40E-03	20	Pdgfb, Casq1, Erbb3, Trdn, Atp2a3, Mylk, Camk2a, Htr2b,

Disease Models & Mechanisms • Supplementary information

			Cacna1s, Mylk2, Casq2, Ryr1, Fgf21, Stim1, Adrb1, Hrc, Mylk4, Fgfr3, Ryr3, Tnnc1
Cell adhesion molecules	5.91E-03	12	Cdh1, Ocln, Cdh2, Cd6, Sdc3, Cd28, Cadm1, Ptprf, F11r, Ncam1, Cldn2, Nrxn3
